# Acoustic cavities in 2D heterostructures

**DOI:** 10.1038/s41467-021-23359-7

**Published:** 2021-06-01

**Authors:** Maxim K. Zalalutdinov, Jeremy T. Robinson, Jose J. Fonseca, Samuel W. LaGasse, Tribhuwan Pandey, Lucas R. Lindsay, Thomas L. Reinecke, Douglas M. Photiadis, James C. Culbertson, Cory D. Cress, Brian H. Houston

**Affiliations:** 1 US Naval Research Laboratory, Washington, DC USA; 2 NRC Postdoctoral Fellow at Naval Research Laboratory, Washington, DC USA; 3 Department of Physics, University of Antwerp, Antwerp, Belgium; 4 Materials Science and Technology Division, Oak Ridge National Laboratory, Oak Ridge, TN USA

**Keywords:** NEMS, Two-dimensional materials

## Abstract

Two-dimensional (2D) materials offer unique opportunities in engineering the ultrafast spatiotemporal response of composite nanomechanical structures. In this work, we report on high frequency, high quality factor (*Q*) 2D acoustic cavities operating in the 50–600 GHz frequency (*f*) range with *f* × *Q* up to 1 × 10^14^. Monolayer steps and material interfaces expand cavity functionality, as demonstrated by building adjacent cavities that are isolated or strongly-coupled, as well as a frequency comb generator in MoS_2_/h-BN systems. Energy dissipation measurements in 2D cavities are compared with attenuation derived from phonon-phonon scattering rates calculated using a fully microscopic ab initio approach. Phonon lifetime calculations extended to low frequencies (<1 THz) and combined with sound propagation analysis in ultrathin plates provide a framework for designing acoustic cavities that approach their fundamental performance limit. These results provide a pathway for developing platforms employing phonon-based signal processing and for exploring the quantum nature of phonons.

## Abstract (web summary)

Here, authors report on acoustic cavities in 2D materials operating in the 50–600 GHz range and show that quality factors approach the limit set by lattice anharmonicity. Functionality expanded by heterogeneities (steps and interfaces) is demonstrated through coupled cavities and frequency comb generation.

## Introduction

Acoustic cavities capable of providing spatial confinement for high frequency (1–10 GHz) elastic waves embody a vital part of optomechanical signal processing^1–6^. The ongoing search for implementations of high quality factor (*Q*) acoustic cavities operating in the extremely high frequency range (EHF, 30–300 GHz) is strongly motivated by the promise that nanomechanical devices invoking quantum behavior^1,2,7,8^ will allow for an extended temperature range suitable for reaching zero-point motion. Coupling EHF mechanical strain to an embedded nanoscale quantum system (e.g., quantum emitter)^9–11^ is another area where the extended frequency range may enable devices suitable for interfacing phonons with two-level systems. However, the low phonon speed that is advantageous for 1–10 GHz devices^3^ (and is much celebrated for enabling small device footprints), becomes a challenge as the operational frequency exceeds ~100 GHz. In this frequency regime, the longitudinal phonon wavelength enters the nanometer size range and the critical dimensions fall beyond the limits of electron-beam-based microfabrication methods, thus necessitating a different approach in cavity design.

Two-dimensional (2D) materials enable the fabrication of single-crystal, suspended mechanical structures as thin as a single monolayer and represent a promising platform for confining EHF longitudinal acoustic (LA) phonons where free surfaces are utilized as acoustic mirrors. The ultrafast mechanical response of transition metal dichalcogenide (TMD) films was shown to include coherent LA phonons in the frequency range extending up to 1 THz^12–15^ as the film thicknesses decrease to a few monolayers. The mismatch in acoustic impedance between clamped exfoliated layers and their underlying substrate allow for trapping of optically-generated elastic waves with quality factors on the order of *Q* ≈5 at frequencies up to 200 GHz^12,14,15^. Significantly longer lifetimes for LA phonons (τ_ph_ = 0.36 ns at 100 GHz) were reported for suspended MoSe_2_ films where radiative acoustic losses were excluded^13^. The corresponding improvement in quality factor (Q_MoSe2 at 100GHz_ ≈ 100) puts 2D material-based cavities on a par with implementations based on acoustic distributed Bragg reflectors (DBR) grown by molecular beam epitaxy (MBE) in systems like GaAs/AlGaAs^16–18^. A drastic reduction in cavity modal volume intrinsic to suspended 2D devices represents a significant advantage over DBR cavity implementations, where limited acoustic contrast attainable for MBE-compatible materials (e.g., Z_GaAs_/Z_AlAs_ = 1.2) causes the elastic strain to extend over 10–20 acoustic wavelengths, even for fine-tuned DBRs (e.g., ref. ^19^). In view of these developments, the fundamental factors that define the highest *Q*-values attainable in 2D-based acoustic cavities become of prime importance, as well as the prospects of engineering the ultrafast mechanical response of composite 2D devices in order to extend acoustic cavity functionality.

In this work, we present an experimental study and theoretical analysis of LA phonon lifetimes in MoS_2_ and h-BN that are chosen as an exemplary set of 2D materials with distinct optomechanical properties. We demonstrate that at room temperature (RT), LA phonon lifetimes attainable in MoS_2_-based EHF acoustic cavities (τ_MoS2_ ≈ 2 ns at 100 GHz, Q ≈ 600) are the highest reported to date for 2D materials and provide a comparison with h-BN based cavities (τ_hBN_ ≈ 0.2 ns). The high spectral purity of MoS_2_ devices allows us to study the effects of a unique structural feature for layered materials —monolayer steps—introduced in cavities as a functionality-enabling heterogeneity. We demonstrate laterally-abutted, step-detuned 2D acoustic cavities that behave independently, while being simultaneously accessible for optical excitation and readout. In compound laminar structures, where the heterogeneity is implemented as a MoS_2_/h-BN interface, we exploit cross-plane strain patterns to build a frequency comb generator with nine overtones in the frequency range extending up to 300 GHz. This concept is further extended to a tri-layer MoS_2_/h-BN/MoS_2_ structure, where we show that the acoustic spectrum of the laminar structure is related to vibrational modes of two vertically stacked MoS_2_ cavities coupled through a more compliant thin h-BN layer. An estimate for the coupling strength of Γ = 47 GHz greatly exceeds the linewidth of the cavity and indicates strong coupling, which offers opportunities in phonon-based signal processing. Finally, the anharmonicity-limited acoustic phonon lifetimes in these systems are evaluated using first-principles density functional theory (DFT) calculations. Given the highly anisotropic nature of 2D materials and the fact that the period of mechanical vibrations of interest is commensurate with the lifetimes of thermal phonons at RT (ωτ_th_ ≈ 1), our atomistic approach provides higher confidence in assessing the fundamental loss contributions in these systems. We use quantum perturbation theory, i.e., Fermi’s golden rule, to determine phonon–phonon scattering rates in which the phonon dispersions and the scattering interactions between phonons are obtained from DFT. These calculations highlight the difference in EHF LA phonon lifetimes between MoS_2_ and h-BN and are in good agreement with measurements, indicating that the performance of RT 2D acoustic cavities in the 100–200 GHz frequency range approaches the fundamental limit.

## Results

### Experimental approach

**Fig. 1:**
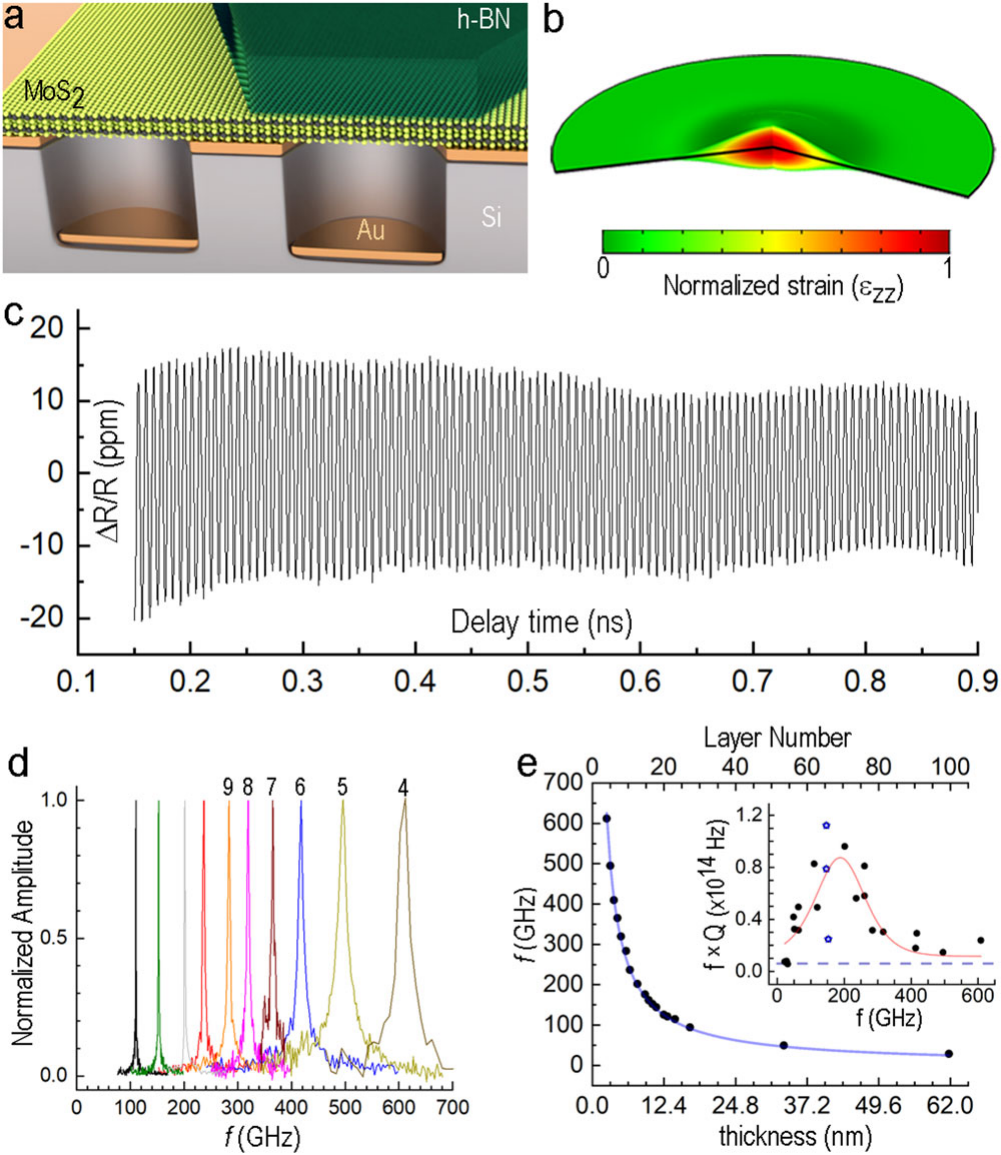
Mechanical response of acoustic cavities implemented as suspended 2D films. **a** Schematic showing MoS_2_ and MoS_2_/h-BN films on a Ti/Au-coated substrate with pre-etched circular wells. **b** Snapshot of the normalized ε_ZZ_ mechanical strain (color coded) in the vibrating part of the MoS_2_ suspended plate as predicted by time-dependent FEM analysis. The pump beam is modeled as Gaussian with radius 0.5 μm. The part of the plate included in the model is limited by the radius 2 μm with low-reflecting outer boundaries. **c** Time-dependent reflectivity of a suspended MoS_2_ film, shown for delay time between 0.15 and 0.9 ns in parts per million (ppm). **d** Examples of normalized FFT spectra of the time-dependent reflectivity taken from different MoS_2_ cavities. The high frequency peaks are labeled with the corresponding MoS_2_ film thicknesses in layer number as extracted from Raman spectroscopy data (see frequency vs. layer number dependence in Supplementary Fig. 2). The peaks are displayed in different colors to help readability. **e** Frequency versus layer number for a range of MoS_2_ acoustic cavities. (inset) *f* × *Q* of MoS_2_ cavities as a function of frequency. The dashed line shows the value required for ground state cooling (6 × 10^12^ Hz). The red curve is added as a guide-to-the-eye and was obtained as a fit using a logistic distribution. Variability in *f* × *Q* values for different cavities in the vicinity of 150 GHz is illustrated by the blue star points with *f* × *Q*_average_ = 0.7 × 10^14^.

The acoustic structures examined here were assembled using a rapidly developing set of fabrication tools^20–22^ that enable the formation of 2D laminates with material quality comparable to MBE films, while alleviating the restrictions intrinsic to MBE (e.g., lattice matching, thermal expansion). MoS_2_ and MoS_2_/h-BN structures were prepared using either one-step direct mechanical exfoliation or sequential stamping and transfer of flakes onto gold-coated substrates with pre-etched wells (see Fig. 1a, METHODS, and Supplementary Figs. 1, 2).

An ultrafast near infrared (NIR) optical pump-probe setup with spatial resolution of about 1 μm was used to evaluate the local, time-resolved mechanical response of the suspended structures. We refer to the portions of the 2D structures undergoing optically-generated thickness-mode vibrations as “acoustic cavities,” and consider these as an extension of the trapped energy resonator concept^23^. Given the semiconducting nature of MoS_2_, the deformation potential is likely to be the dominant mechanism for generating optically-induced elastic strain^24,25^. The large penetration depth for the pump laser (770 nm) in MoS_2_ ensures uniform excitation and thus homogeneous initial stress along the normal component. Optical readout for the mechanical response is provided by delayed probe pulse (830 nm wavelength), as its reflectivity is modulated by both film dilation (Fig. 1b) and photoelastic effects^26,27^, also magnified by interferometric effects via a Fabry–Perot optical cavity (see METHODS). Finite element modeling (FEM)^28^ was used to analyze and to interpret the outcome of pump-probe experiments. Calculations fully account for the anisotropy of bulk TMD materials and h-BN. In accord with Greener et al.^15^, continuum elasticity-based models were consistent with the experimental results in the sub-THz frequency range.

### MoS_2_ acoustic cavities

The normalized reflectivity (ΔR/R) of a suspended MoS_2_ film, after subtracting a slowly varying background, is shown in Fig. 1c as a function of delay time between the pump and probe laser pulses (raw data is shown in Supplementary Fig. 3). A pronounced modulation is attributed to the thickness-mode vibrations since the single peak in the FFT spectrum (e.g., Fig. 1d) matches the expected resonance frequency for a sound wave where a half-wavelength fits across the film thickness (146.25 GHz for the 18-layer (18 L), 11.2 nm film in Fig. 1c). The variation of the FFT spectra with the film thickness measured in number of monolayers *N* (0.62 nm per layer for MoS_2_^29^) is shown in Fig. 1e and follows the expected trend of *f ∼N*^−1^ (see for example ref. ^13^). Fitting the data in Fig. 1e provides an average value for the cross-plane LA sound speed *c*_LA_ = 3170 m/s in our MoS_2_ films.

The slow decay of the pump-induced vibrations (Fig. 1c) indicates minimal energy losses and highlights a low internal friction of crystalline MoS_2_, as well as negligible “phonon leaking” (caused by lateral spreading of pump-driven elastic excitations) in the 100 GHz-range TMD cavities. A ring-down time of τ ≈ 1.6 ns that is extracted via fitting an exponentially-decaying time dependence to the vibrational amplitude in Fig. 1c corresponds to a cavity quality factor *Q* = 730 at *f*  = 146.25 GHz. For this cavity, the resulting *f* × *Q* = 1.1 × 10^14^ Hz is the highest measured in our experiments and also the highest reported to date for a 2D material system (to our knowledge). The physical mechanisms that define the fundamental limits attainable for *Q* values (i.e., phonon lifetimes) in different 2D materials will be discussed below. While differences in the performance of individual cavities can be 5× (attributed to variability in manual exfoliation), the *f* × *Q* product for our MoS_2_ devices remains at or above that required for ground state laser cooling (*f* × *Q* = 6 × 10^12^ Hz)^30^. Figure 1d, e inset highlight the frequency dependence of energy losses and suggest that the 100–200 GHz frequency range is favorable for maximizing the *f*  × Q product in this measurement geometry at RT. Therefore, we focus on the corresponding range in film layer number, centered at about 10 nm (*N* ~16 layers), and demonstrate that the layered nature of 2D materials enables introduction of well-controlled heterogeneities that can alter the underlying elastic-strain patterns and expand cavity functionality by modifying the ultrafast mechanical response.

### Step-detuned cavities in MoS_2_

**Fig. 2:**
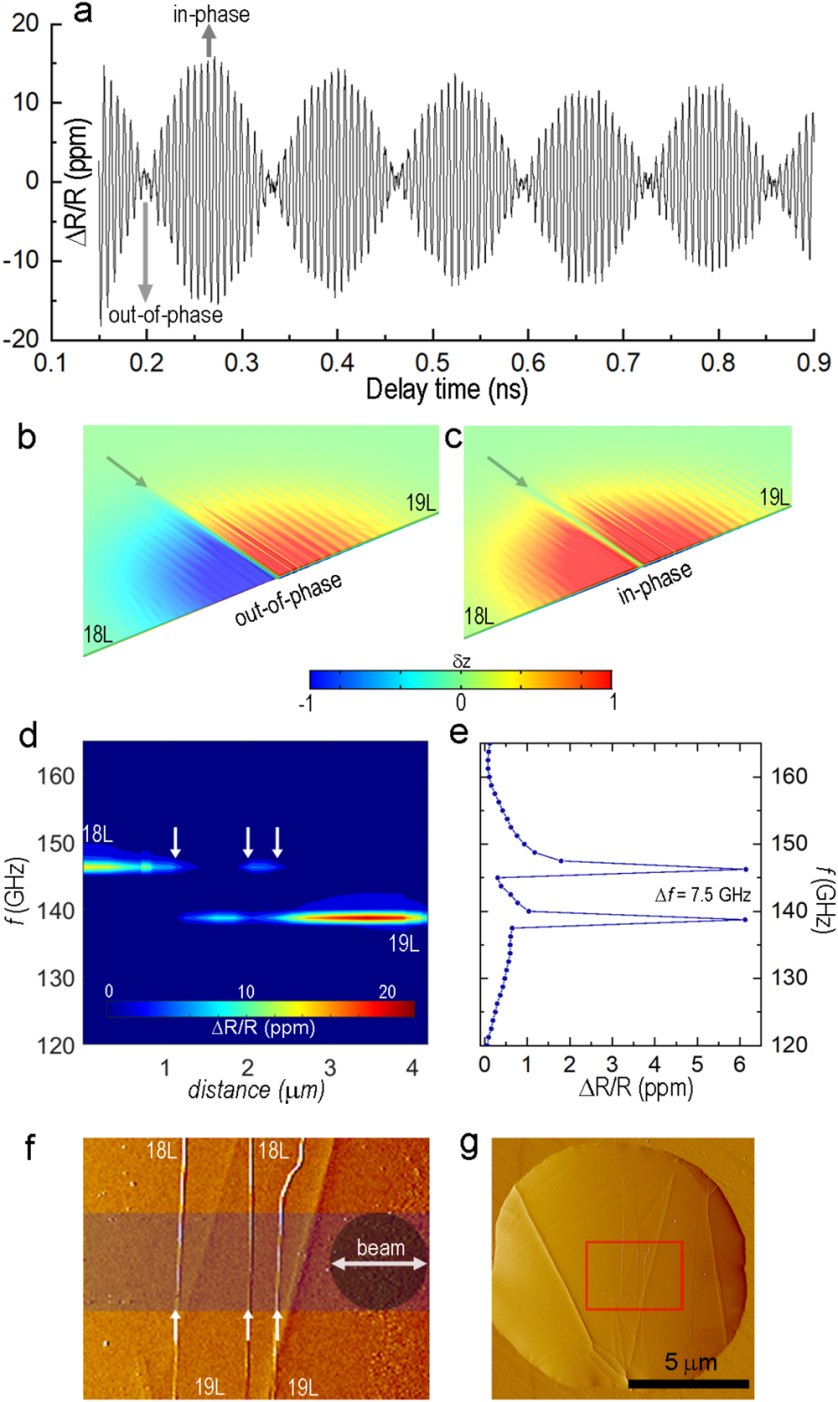
Horizontally-abutted cavities divided and detuned by a monolayer step. **a** Time-dependent reflectivity taken at a monolayer step boundary on an 18-layer (18 L)/19-layer (19 L) MoS_2_ suspended film. The in-phase and out-of-phase labels show the timing for the snap-shots in **b**, **c**. FEM-generated snapshots of normalized vertical displacement maps (δz) highlighting the out-of-phase (**b**) and in-phase (**c**) vibrations of the abutted cavities at the monolayer step. Additional information for the time-domain FEM analysis are included in the Supplementary Information (Supplementary Note 2 and Supplementary Movie 1). **d** 2D plot showing color-coded FFT spectra of the probe reflectivity acquired at different positions while stepping the beam across the monolayer steps shown in **f**. **e** FFT spectrum of time-dependent reflectivity from **a**, which is taken approximately at the 2.2 μm position in **d**. **f** AFM phase-image showing the region where the spectra in **a** and **d** were acquired. The monolayer steps run vertically across the image. The horizontal darkened band shows the path where the 1 μm diameter beam was scanned across the surface. **g** Low magnification AFM phase-image showing the MoS_2_ drum (see Supplementary Fig. 4 for optical image). The red box highlights the region imaged in **f**.

Toward the goal of employing heterogeneities as acoustic tools, we consider discrete thickness variations, or steps, between atomically-flat regions on a 2D material surface as a reproducible and well-controlled feature for altering the ultrafast response. The high *Q* value of our MoS_2_ cavities allows us to resolve perturbations in the mechanical response of 100 GHz devices induced by the presence of even a single monolayer step. Figure 2a shows the time-dependent reflectivity acquired for a suspended MoS_2_ film with a 1 L step positioned at the center of the coinciding pump/probe beams (optical image in Supplementary Fig. 4).

Pronounced beats observed during the ring-down (Fig. 2a) manifest as two distinct peaks in the FFT spectrum (see below), in striking contrast to the monochromatic response of homogeneous cavities in Fig. 1d. The split Δf ≈ 7.5 GHz between the experimentally observed peaks in the spectrum of the “step-cavity” closely matches the detuning expected from a monolayer change in thickness and suggests that the excited parts of the film on each side of the step behave as co-vibrating, but nearly isolated acoustic cavities. Such interpretation implies that the experimentally observed beats in the intensity of the reflected probe pulse arise when the photodetector sums up the contributions from two concurrent but frequency-shifted vibrational modes (Fig. 2b, c). If we assume that some degree of coupling is present between the half-cavities, these vibrational modes could be considered as mixed modes (ω_±_) of coupled resonators (see for example ref. ^31^ and Supplementary Note 1). In the weak coupling limit κ_coupling_ → 0, the frequencies of the mixed modes are expected to approach those corresponding to flat plates (18 L and 19 L).

To evaluate the coupling strength, we compare the vibrational spectra acquired on the step with the response far away from the step. Figure 2d shows a filled contour plot of the spatially varying FFT spectra (see example of a single spectra in Fig. 2e) as coinciding pump/probe beams are moved laterally across the monolayer steps imaged in Fig. 2f, g. Regardless of the measurement position (“on step” or “away from step”) we only observe a set of discrete frequencies, each corresponding to a whole number of MoS_2_ monolayers. There is no evidence of frequency deviation for the mixed modes in the vicinity of the steps, at least not within the signal-to-noise ratio available in our experiments. This result indicates that despite the cavities being formed mostly by the same atomic planes, the lateral coupling between the abutted cavities does not exceed the halfwidth of the resonance (≈0.2 GHz). Employing a one degree-of-freedom (1DOF) model for two coupled and nearly identical oscillators^31^ (Supplementary Equation 3), this result provides an upper boundary estimate for the spring constant ratio κ_coupling_/k_oscillator_ ≈ 2 × 10^−3^, implying nearly independent cavities. This estimate confirms that in contrast to “acoustic molecules”^32^, the energy transfer between laterally abutted 2D cavities is negligible, at least within the pump repetition period in our experiments. Therefore, the low frequency envelope in Fig. 2a provides the frequency difference between the abutted half-cavities (ω_+_–ω_−_, Fig. 2a), where each can be viewed as independent. We emphasize that such a differential readout is enabled by the nature of well-defined monolayer steps in 2D materials and note that recently developed transfer methods allow for controlled placement of such steps in top-down lithographical approaches (e.g., adding a monolayer film on half of a uniformly thick slab^33^).

Simultaneous optical response from the adjacent acoustic cavities can enable frequency down-conversion, where one of the half-cavities acts as a local oscillator for the other half-cavity. Recent progress in developing nonlinear optical detectors^34^ opens the possibility for mixing intensity-modulated optical signals coming from the abutted-cavities directly at the photodetector. An estimated bandwidth of ~0.5 THz for the graphene-based mixer-detector^34^ could allow real-time down-conversion with the output electric signal from the photodetector modulated at the beat frequency of adjoined TMD cavities. We also note that although our experiments do not show cyclic energy exchange between the half-cavities, a step on the area of the plate that undergoes thickness-mode vibrations can generate a steady energy outflow (i.e., leakage) by launching slow propagating longitudinal elastic waves known as Lamb waves^23,35^. These waves are manifested as the ripples seen in Fig. 2b, c and for a nanostructure embedded in the plate, they can make a remote, high-*Q*, pulse-driven step-cavity to appear as a narrow-band Lamb wave generator with the frequency defined by the plate thickness. While beyond the scope of this work, this concept can open opportunities for in-plane (“on-chip”) EHF acoustics. Unfortunately, within our experimental approach, these effects are muted by the disparity between the lateral extent of the diffraction-limited probe laser spot and the much shorter in-plane wavelengths of step-generated elastic excitations.

### h-BN/MoS_2_ bilayer: Frequency comb and h-BN phonon lifetime

To provide stronger coupling for our optical readout system, we explore heterogeneities that span the full laser spot diameter, such as those formed at the boundaries of dissimilar materials in laminar structures. Modifications to an acoustic cavity that enable access to high-frequency overtones can be implemented by invoking highly nonuniform strain patterns for the cavity excitation. To ensure such a nonuniformity in a laminated stack of 2D materials, we assembled devices composed of a relatively thick h-BN layer (44 nm) van der Waals (vdW) bonded to a thinner MoS_2_ layer (8.7 nm or 14 L; optical image in Supplementary Fig. 4). The large disparity in material bandgaps (h-BN ≈ 6 eV and bulk MoS_2_ ≈ 1.2 eV) limits optical absorption of the pump beam to the MoS_2_ layer alone, thereby making the TMD a thin transducer that drives the full 53 nm thick stack.

**Fig. 3:**
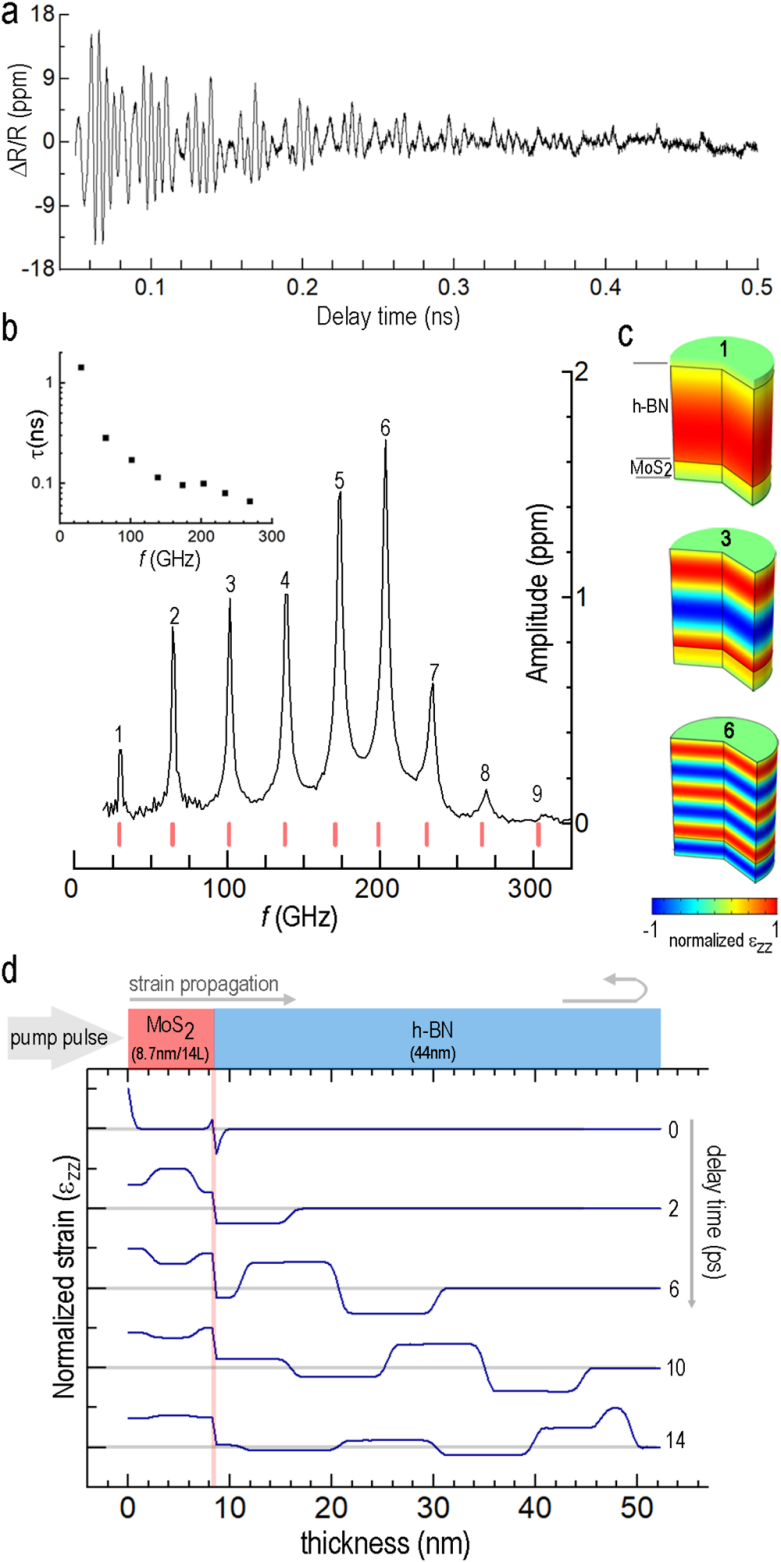
Frequency comb generator implemented in a h-BN/MoS_2_ bilayer. **a** Time-dependent reflectivity for a h-BN (44 nm)/MoS_2_ (8.7 nm/14 L) suspended film. **b** FFT spectrum of the signal shown in **a**. The red tick marks along the bottom of the plot show positions of the bilayer overtones as predicted by FEM eigenfrequency analysis. The peaks are numerically labeled from lowest to highest frequency. (inset) Extracted lifetimes for h-BN (*τ*_hBN_) versus frequency for peaks in **b** (see Supplementary Note 4 for details of the extraction method). **c** Spatial configuration of the normalized ε_ZZ_ strain for three different modes (labeled) in the h-BN/MoS_2_ heterostructure (eigenfrequency FEM analysis, 1D model). **d** Snapshots of the ε_ZZ_ strain distribution generated by optically-excited MoS_2_ are shown across the thickness of the bilayer at early delay times (for details of time-domain axisymmetric FEM analysis, see Supplementary Note 3).

Figure 3a shows the time-dependent reflectivity from a h-BN/MoS_2_ heterostructure, which is notably different from both the homogenous cavities in Fig. 1 and the “step-cavities” in Fig. 2. Despite the complex appearance, the temporal behavior is straightforward to interpret in the frequency domain, where a pronounced frequency comb emerges with a series of nearly equidistant peaks with separation Δ*f* ≈ 34 GHz (Fig. 3b). Using eigenfrequency numerical analysis, we find that the center frequency for each experimentally observed peak coincides with that of corresponding high-order overtones (red ticks) in the laminated h-BN/MoS_2_ structure. Examples of the spatial configuration of the strain for the high-order overtones can be seen in Fig. 3c. The frequency comb extends to the ninth-order overtone and spans nearly four octaves, which is unattainable for a uniformly excited homogeneous film (e.g., Fig. 1d). The larger amplitudes of high-order overtones in Fig. 3b are directly related to the highly inhomogeneous strain generated across the h-BN/MoS_2_ stack by the pump pulse. Figure 3d shows snapshots of strain patterns calculated using time-domain FEM analysis (see Supplementary Note 3 for details of the modeling approach). Expansion of the MoS_2_ film is driven by the deformation potential^25^ and generates a succession of nearly rectangular strain pulses launched into the h-BN layer, which then propagate at the speed of the longitudinal sound waves (*c*_LA_ ≈ 3.4 × 10^3^ m/s for h-BN; the snap-shots of the spatial distribution for the out-of-plane strain in the bilayer are shown in Supplementary Figs. 6, 7 and Supplementary Movie 2). The envelope of the comb in the experimentally acquired spectrum (Fig. 3b) peaks at about 200 GHz and is defined by the sample geometry. An overtone is prominent when the wavelength of the standing wave, *λ*_s_ (see Fig. 3c) closely matches that of the elastic wave launched by the MoS_2_ transducer in response to pulse excitation (Fig. 3d). Here, the sixth overtone (~200 GHz, strain pattern in Fig. 3c) has the closest overlap since the MoS_2_ layer thickness approaches the half-wavelength, *λ*_s_/2. Accordingly, the inverse round-trip time for sound within the MoS_2_ is commensurate with the overtone’s frequency.

The bilayer h-BN/MoS_2_ geometry used here provides a point of comparison with a similar transduction approach based on spatially-inhomogeneous optical absorption that has been used to generate a broadband frequency comb in GaAs/AlGaAs/InGaAs systems^36,37^. A distinct advantage offered by 2D materials is the higher level of spatial confinement, which is illustrated by comparing our 53 nm thick h-BN/MoS_2_ stack versus the combined 407 nm thick multiple quantum well structures (plus 3.76 μm for the DBR reflector) in ref. ^37^. More importantly, given the wide frequency range of excited overtones, our measurement provides experimental evaluation for the frequency dependence of the longitudinal phonon lifetimes in h-BN (Fig. 3b inset), as the energy dissipation in our bilayer structure can be separated into individual contributions from h-BN and MoS_2_ (see for example ref. ^38^ and Supplementary Note 4 with the elastic energy distribution for different vibrational modes in the bilayer shown in Supplementary Fig. 8, and energy partitioning coefficients listed in Supplementary Table 1). These results are enabled by the high-quality, low-loss MoS_2_ transducer layer since h-BN alone would be inaccessible for a NIR wavelength pump-probe setup. While a metal transducer evaporated on a semiconductor film can also be used to generate a frequency comb^39,40^, the use of a crystalline 2D material-based transducer with low internal friction and diminished surface roughness can reduce the overall energy losses and make the estimates for the phonon lifetimes more reliable. Importantly, a wide variety of high-contrast bilayer structures with atomically-sharp interfaces can be built using 2D materials. The transfer approach mitigates problems inherent to physical vapor deposition, such as island growth modes (due to dissimilar surface energies) or interdiffusion, as well as built-in stress associated with many evaporated/epitaxial films. Our control experiments with stacked MoS_2_/MoS_2_ slabs indicate that the adopted method for flake transfer, including exposure to ambient air does not increase the mechanical losses considerably (see Supplementary Note 5 and frequency dependence of the quality factor *Q* for MoS_2_/MoS_2_ stacks shown in Supplementary Fig. 9). Similar low-loss behavior is projected on MoS_2_/h-BN boundaries based on transmission electron microscopy (TEM) results that find pristine interfaces between h-BN and MoS_2_^41^. Close values of interface adhesion energy (0.3 J/m^2^ for MoS_2_/h-BN versus 0.4 J/m^2^ for MoS_2_/MoS_2_ interfaces) also imply commonalities in vdW self-cleansing mechanism for these interfaces^42^ and therefore, similar losses.

### Coupled cavities in MoS_2_/h-BN/MoS_2_ tri-layer

In the implementation of the frequency comb described above, we centered our analysis on energy dissipation within individual cavity components and on relative intensities of the excited overtones in the bilayer h-BN_(44 nm)_/MoS_2 (8.7 nm)_ cavity. Building on this assessment, we extend the multilayer design approach in an effort to reengineer the cavity vibrational modes themselves. To illustrate mode alteration, we sandwich a thin h-BN film between two MoS_2_ layers (MoS_2(17L)_/ h-BN_(5L)_/ MoS_2(15L)_) in an effort to tailor the strain patterns and the corresponding spatial redistribution of the elastic energy across a tri-layer laminar structure (optical images of the tri-layer structures and elastic energy distribution for selected vibrational modes are shown in Supplementary Figs 10, 11).

**Fig. 4:**
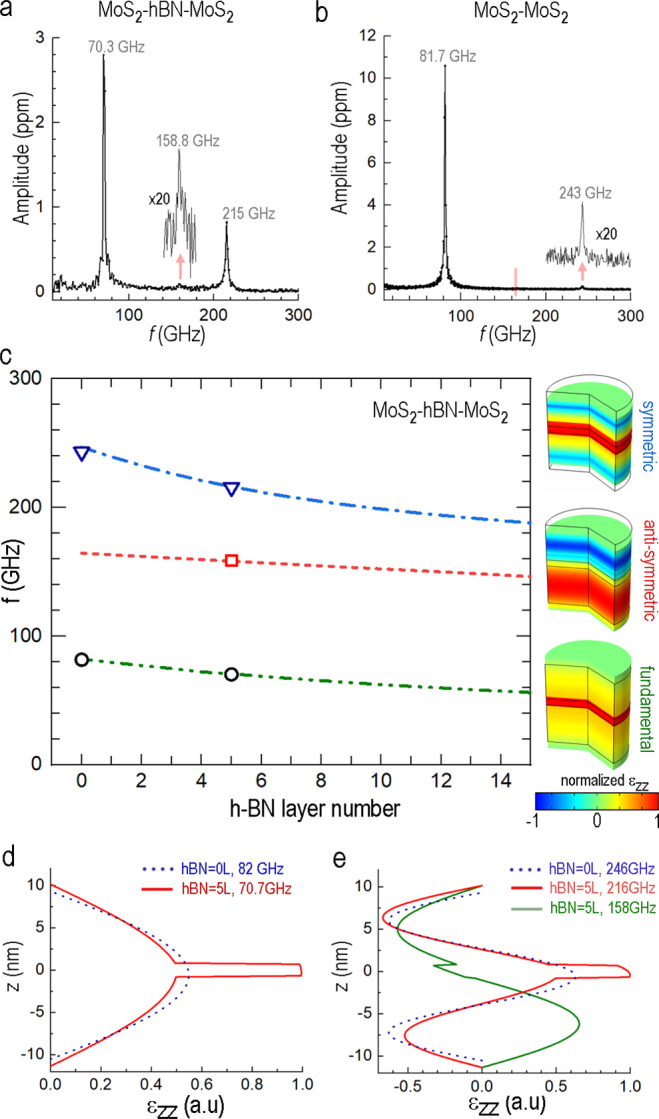
Strongly coupled acoustic cavities constructed as a vertically-stacked heterostructure. **a** Spectrum for the MoS_2_/h-BN/MoS_2_ tri-layer; **b** Spectrum of the neighboring MoS_2_/MoS_2_ control area. The red tick in **b** highlights the location of the non-active second harmonic peak. Only odd modes are excited with the homogeneous pump excitation here. **c** FEM-predicted frequencies for the three lowest vibrational modes of the tri-layer system as function of the spacer h-BN layer thickness. Experimental data (open symbols) are included for the tri-layer (h-BN thickness = 5 L, normalized ε_ZZ_ strain configurations are shown on the right) and for the control sample (h-BN thickness = 0 L). **d**, **e** The normalized ε_ZZ_ strain profiles for vibrational modes in monolithic MoS_2_ (dotted lines) are shown in comparison with strain in the tri-layer system (solid lines) at the labeled frequencies.

Figure 4 compares the measured vibrational spectrum for a tri-layer MoS_2(17L)_/h-BN_(5L)_/ MoS_2(15L)_ sample (Fig. 4a) with the spectrum of a control bi-layer MoS_2(17L)_/ MoS_2(15L)_ sample (Fig. 4b). A significant increase in the intensity of the third overtone (215 GHz, Fig. 4a) is a notable outcome of introducing the h-BN middle layer. However, even more important is the fact that the position of the second overtone in the tri-layer structure (158 GHz, Fig. 4a) becomes far detuned from the doubled frequency of the fundamental mode (70.3 GHz). The fundamental mode frequencies for both devices match the FEM-predicted values (Fig. 4c), which provides confidence that the material interfaces behave as “ideal boundaries” with no softening due to interlayer contamination^15^. Dotted lines in Fig. 4c show the calculated frequencies for the first three vibrational modes of the tri-layer stack as function of the h-BN thickness. The salient feature is the progression from equidistant harmonics in the monolithic MoS_2_ film (at h-BN = 0 nm) to a set of second and third overtones (red and blue lines, Fig. 4c) that converge towards the resonant frequency for an isolated 17L-thick MoS_2_ layer (154 GHz). Such a tendency is governed by the strain patterns arising from the presence of the h-BN layer (Fig. 4d, e). For the second overtone (anti-symmetric, ≈ 158 GHz) the location of h-BN layer coincides with the strain node. As a result, the reduced material stiffness (C_33_ = 27 GPa in h-BN^43^ versus C_33_ = 54 GPa in MoS_2_^44^) becomes inconsequential in the elastic response. On the contrary, the third overtone (symmetric, 216 GHz) applies maximum strain to the h-BN layer, which leads to a reduced restoring force and lower resonant frequency. The overtones moving closer to each other invoke a model where two MoS_2_ slabs can be considered as distinct acoustic cavities coupled through the h-BN barrier. The third overtone is then interpreted as *ω*_+_ (symmetric) mode, while the second overtone is assigned as *ω*_−_ mode (see refs. ^30,31^ and Supplementary Notes 1, 6). Experimental data for the overtones’ positions in our tri-layer stack are shown by open symbols in Fig. 4c and demonstrate a clear step toward a coupled-cavity implementation. A 1DOF model for two coupled mechanical oscillators (see Supplementary Equations 3, 4) applied to our tri-layer system produces coupling strength Γ ≈ 47 GHz and κ_coupling_/k_oscillator_ ≈ 0.35, implying very strong coupling.

Apart from the interplay between the high-frequency overtones, the fundamental vibration mode in our tri-layer system is of prime interest since it features a large and nearly uniform strain profile within the h-BN layer (Fig. 4d). Known to be a good host for RT quantum photon sources^45–48^, the h-BN spacer layer sandwiched between MoS_2_ “hammer and anvil” represents a promising configuration for coupling embedded optical emitters to coherent LA phonons confined in the tri-layer cavity. Importantly, both the maximum attainable strain, as well as the spectral purity of the elastic stimulus delivered to embedded nanostructures are defined by the quality factor of the overall cavity. Energy losses in both the h-BN “device layer” and surrounding MoS_2_ structures affect the total phonon lifetime in the cavity. The experimental values for the tri-layer ring-down time τ_Total_ = 0.65 ns fall between our previously extracted values for τ_hBN_ = 0.24 ns and τ_MoS2_ = 2.8 ns at 70 GHz and agree to within 10% with the prediction 1/τ_Total_ = α_1_/τ_hBN_ + α_2_/τ_MoS2_ where α_1,_ α_2_ are energy partition coefficients for the tri-layer structure (see Supplementary Table 2). We view this agreement as a strong indicator that losses related to the MoS_2_/h-BN interfaces do not limit the phonon lifetime in the cavity.

## Discussion

Developing a toolbox for building high-performance acoustic cavities in 2D materials requires a quantitative understanding of the fundamental mechanisms that govern phonon lifetimes, including both frequency and temperature dependences. It is also necessary to account for both material-specific internal friction, as well as extrinsic effects (e.g., surfaces and imperfections, confinement losses, etc.). Intrinsic losses in the nonmetallic materials studied here (at RT) arise mainly from phonon–phonon scattering, often referred to as anharmonic effects. Until recently only semiquantitative models, developed primarily for sound attenuation in isotropic materials and intended for frequency limits ωτ_th_ << 1 or ωτ_th_ >> 1, have been available for fitting measured data (ω is the frequency of the sound wave and τ_th_ is the lifetime of thermal phonons, see for example refs. ^49,50^). Advancements in the development of first-principles techniques now allow for fully microscopic treatments of phonon lifetimes^51,52^ (see Supplementary Notes 7, 8 for description of theoretical approach), which do not invoke uncertainties resulting from experimentally-derived empirical constants within the model (e.g., average Grüneisen parameters). Relatively little work of this kind has been done in predicting phonon lifetimes for low frequency acoustic phonons^51,52^, particularly in comparing with measured data in low dimensional and vdW layered systems.

**Fig. 5:**
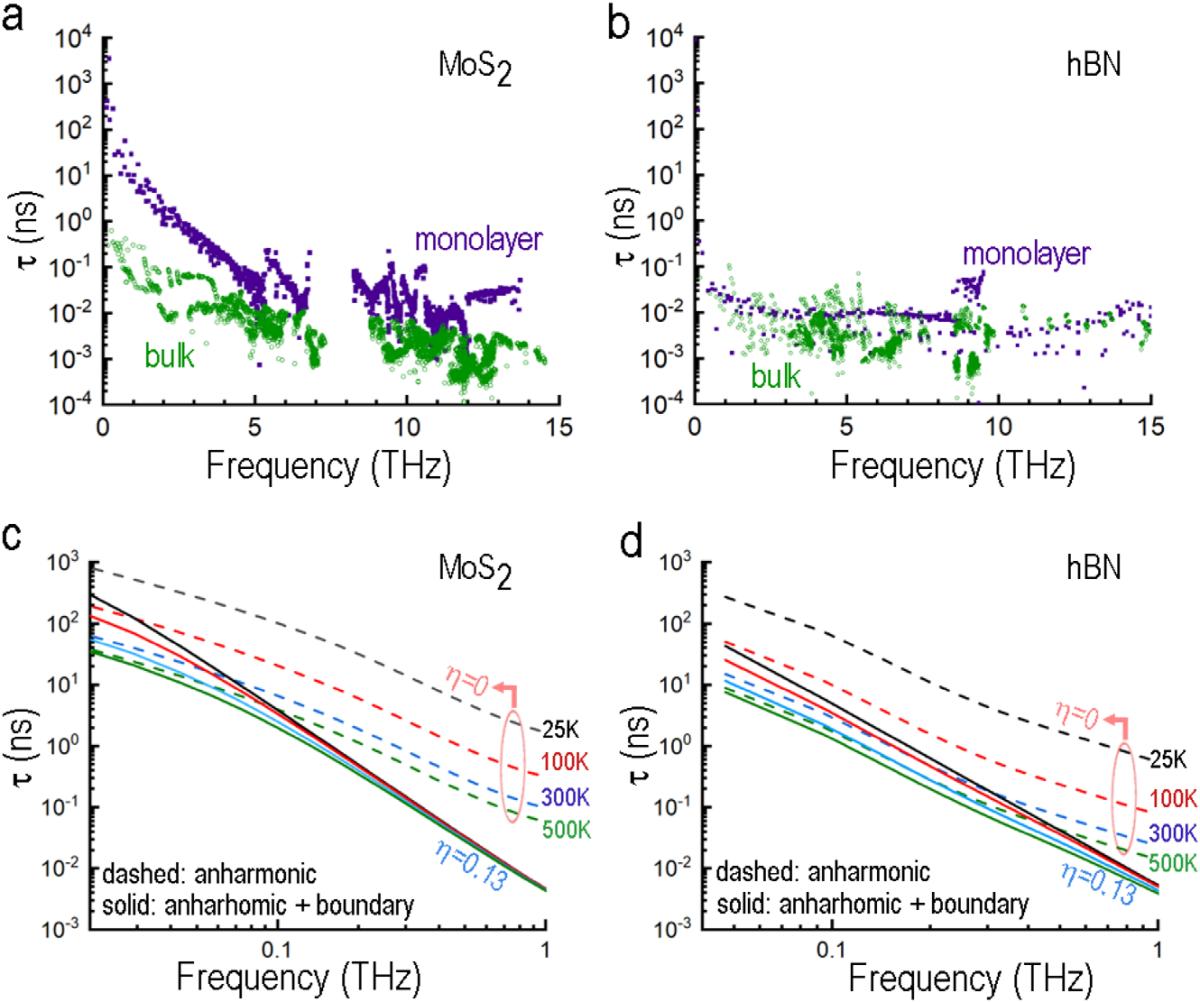
Phonon lifetimes due to anharmonic phonon–phonon scattering for bulk and monolayer systems. Phonon lifetimes through the Brillouin zones for all polarizations and propagation directions at room temperature in **a** MoS_2_ and **b** h-BN. For h-BN, phonon lifetimes are truncated at 15 THz. Lifetimes over the entire frequency range are shown in Supplementary Fig. 16. Low-frequency plot (log-log) showing temperature dependences of lifetimes for cross-plane LA phonons in **c** MoS_2_ and **d** h-BN from anharmonic scattering (dashed curves, η = 0) and from the sum of anharmonic scattering and surface losses (solid curves, η = 0.13). *η* is the root mean square variation of the surface height (i.e., rms roughness in nanometers).

Our calculated phonon lifetimes for all polarizations due to anharmonic three-phonon scattering alone through the Brillouin zones for bulk and monolayer MoS_2_ and h-BN are shown in Fig. 5a, b (see METHODS; also see Supplementary Figs. 12–14 for schematics of the theoretical setup and Supplementary Figs. 15, 16 for phonon lifetimes projected onto the phonon dispersion curves, as well as full Brillouin zone data). They vary widely in magnitude across each Brillouin zone and become considerably longer at low frequencies. For thermal phonons (*f*_th_ = *k*_B_*T*/(2 *π ħ*) ≈ 6.25 THz at RT) the predicted lifetime is close to τ_th_ ≈ 1–3 ps for both bulk h-BN and MoS_2_. The ability to cover seamlessly the entire low frequency range (*ħω* < *k*_B_*T*), including the crossover at *ω*_c_*τ*_th_ ≈ 1 (which is of prime interest here at *ω*_c_ ≥ 2*π* × 55 GHz), is a major advantage of our DFT-based approach. The lifetimes in MoS_2_ and MoSe_2_ (see Supplementary Figs. 15, 16) were found to be similar in overall magnitude, and those in h-BN are somewhat shorter, especially near the *f* ≈ 1 THz range. To illustrate the versatility of the microscopic approach, we also present the phonon lifetimes calculated for monolayers of MoS_2_, h-BN and MoSe_2_ (see Supplementary Fig. 16). The enhanced phonon lifetimes predicted for the TMD monolayers MoS_2_ and MoSe_2_ (though not so for h-BN) suggest that the intrinsic anharmonic losses are less significant in these materials as layer thicknesses are reduced. At lower frequencies in particular, out-of-plane flexure vibrations with quadratic dispersions in monolayer materials tend to have long lifetimes. These modes are not present in their bulk counterparts.

In order to represent acoustic vibrations in films with varying thicknesses *h* we use calculations for bulk LA modes polarized perpendicular to the surfaces with wave vectors *q*_z_ and with half wavelengths equal to the layer thicknesses, *h* = *λ*_z_/2 = *π*/*q*_z_. Their frequencies as functions of thickness are found to agree well with the corresponding measurements in Fig. 1e. The phonon lifetimes due to anharmonic phonon scattering alone in the THz regime for MoS_2_ and h-BN are given by the dashed curves in Fig. 5c, d, where they are seen to decrease with frequency. The temperature dependences of the anharmonic contributions to phonon lifetimes for MoS_2_ (Fig. 5c) and h-BN (Fig. 5d) are pronounced and arise from the phonon population factors in the phonon–phonon scatterings. Lower temperatures can reduce anharmonic scattering losses considerably.

To represent the extrinsic contributions to the vibration lifetimes, a simple model for surface losses due to scattering from randomly varying surface or boundary heights^53^ is used (solid lines Fig. 5c, d). This gives a scattering contribution in the form

1 $$1/{{\tau }}_{{{\mathrm{surface}}}}=\frac{1-p}{1+p}\frac{\left|{v}_{z}\right|}{h},p={e}^{-{\left(4{\pi }{\eta }/{{\lambda }}_{z}\right)}^{2}}$$

where *p* is the specularity of the surface, *v*_z_ is the group velocity, λ_z_ is the acoustic wavelength and *η* is the root mean square variation of the surface height (i.e., rms roughness). Recent measurements on freshly cleaved MoS_2_ surfaces give an estimated *η* = 0.23 nm^54^, which could be even smaller for clean MoS_2_ surfaces with fewer sulfur vacancies and adsorbed species.

**Fig. 6:**
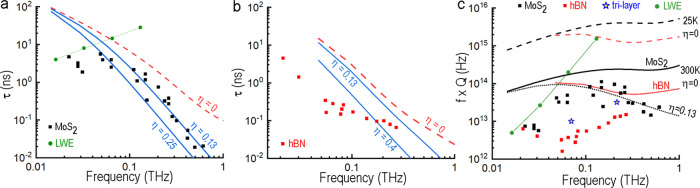
Experimental and theoretical results for the lifetimes of LA phonons polarized perpendicular to the basal plane. Frequency dependence predicted for the room temperature phonon lifetimes in **a** MoS_2_ and **b** h-BN as defined by anharmonic scattering alone (dashed curves; η = 0) and by the combination of anharmonic scattering with surface losses (solid curves; η > 0) for two values of rms surface roughness (η) in nanometers. Values extracted from experimentally measured cavity ring-down times are shown by the black (**a**) and red (**b**) square points. The green points in **a** and **c** show the FEM-calculated time for Lamb waves escape LWE (see Supplementary Note 9 for details). **c** Comparison of calculated and measured *f* × *Q* product as a function of frequency. The black lines show *f* × *Q* values calculated for MoS_2_ cavities and the red lines correspond to h-BN structures at the labeled temperatures and η. Experimental data for cavities studied in the present work are given by the square and star symbols as labeled. The tri-layer data points represent the MoS_2_/h-BN/MoS_2_ heterostructure (see Fig. 4).

To assess how well these microscopic calculations compare with our measured data, Fig. 6a shows the experimental ring-down times for the monolithic MoS_2_ cavities described earlier (see Fig. 1). The sum of the anharmonic and surface roughness losses are in very good agreement with experiment for the frequencies *f* ≥ 100 GHz, both for magnitude and for frequency dependence. Two different values of *η* are provided to illustrate the effects of varying surface roughness. The two solid blue curves in Fig. 6a give the sum of these surface losses and the anharmonic scattering for MoS_2_ for *η* = 0.25 nm and for *η* = 0.13 nm, where the latter is fit to the measured data here (black squares).

A broad frequency range comparison of our microscopic calculations and experimental data against the well-accepted models for absorption of sound in insulators^55^, based on Landau–Rumer and Akhiezer approach, shows that ab initio predictions for τ in the intermediate range (i.e., ωτ_th_ ≈ 1) are an order of magnitude larger (see Supplementary Fig. 17 for the comparison of ab initio calculations, experimental data, and asymptotic theoretical models). We emphasize that in the absence of the DFT-based results any quantitative analysis of dissipation above 50 GHz (ωτ_th_ ≈1) would be challenging and assumption-driven (see for example ref. ^50^). That frequency range is of prime importance here, since at 100 GHz the performance of our MoS_2_-based cavities is within a factor of three from the fundamental limit defined by the intrinsic anharmonicity-based losses (Fig. 6a). Unexpectedly, the experimental ring-down times deviate down from our theoretical predictions at *f* < 100 GHz. While some limitations of the measuring technique can be a contributor, we attribute most of this divergence to another extrinsic loss mechanism only recently considered in ultrathin photoexcited semiconductor plates^56^ —the lateral spreading of pump-generated elastic waves that propagate outward from the probe-beam spot as Lamb waves^23^ (see Supplementary Note 9). The escape rate of these waves, as opposed to a phonon lifetime, can be a limiting factor in the experimentally-measured ring-down at lower frequencies. Given the absence of any structurally-defined lateral confinement in our films (e.g., walls or steps), the escape time is governed by the details of Lamb wave dispersion (see Supplementary Note 10) and scales approximately as a ratio of pump spot size, *R*_pump_, to film thickness, *h*, τ_Lamb_ ~ *R*_pump_/*h*, which makes the runaway more pronounced for thicker films. The analytical approach developed by Photiadis et al.^56^ is consistent with FEM modeling for MoS_2_ films (see Lamb wave dispersion calculated using eigenfrequency analysis and ring-down time extracted from time-domain simulations in Supplementary Figs. 18, 19). The estimates for the Lamb waves escape times (LWE) produced by numerical modeling are shown by green points/line in Fig. 6a and agree well with the low frequency leveling-off of our experimentally measured ring-down times.

It is evident from comparing Figs. 1c and 3a that the ring-down time measured for h-BN-based structures is significantly shorter than that exhibited by monolithic MoS_2_ films. Figure 6b shows that the phonon lifetimes in h-BN (extracted from Fig. 3b and additional h-BN/MoS_2_ cavities) is consistently lower than the theoretical limit defined by phonon–phonon scattering. The dashed line in Fig. 6b is for anharmonic phonon scattering alone (*η* = 0) while two solid curves show the cumulative effects of phonon–phonon as well as boundary scattering. Comparison with the experimental data shows that even the upper-bound value for h-BN surface roughness reported in the literature *η* = 0.1–0.4^57,58^ does not fully account for the extra dissipation in our devices. The presence of the h-BN/MoS_2_ interface cannot explain the extra dissipation since the ring-down times from our tri-layer structure MoS_2_/h-BN/MoS_2_ (which contains two identical interfaces with a very thin h-BN layer) is actually longer than the decay in the h-BN/MoS_2_ bilayer with a single interface (Fig. 6c, blue stars). This discrepancy implies the presence of an additional dissipation mechanism, possibly related to inter-mode coupling or material quality. We anticipate that future low temperature measurements will provide insights into various loss mechanisms in h-BN.

Finally, to gain a broader picture of how these results project the ultimate performance of 2D material-based acoustic cavities, we employ *f* × *Q* products as a figure of merit. Figure 6c compares experimental data with the *f* × *Q* values calculated for bulk MoS_2_ and h-BN at two different temperatures. For anharmonicity-limited systems the predicted *f* × *Q* product increases slightly with frequency (in a qualitative agreement with the Landau–Rumer model^55^), and for a given material represents the highest achievable performance for an acoustic cavity. With the boundary scattering term included for MoS_2_ (η = 0.13) the *f* × *Q* product decreases with increasing frequency (dotted line Fig. 6c), and fits our experimental *f* × *Q* values for MoS_2_ (black squares Fig. 6c). The order of magnitude increase in *f* × *Q* projected at low temperature is highly desirable, but will only become attainable if boundary scattering can be mitigated (see for example ref. ^21^).

The presence of a “goldilocks zone” centered near *f* ≈ 100–200 GHz, where currently available materials and fabrication techniques yield the highest *f* × *Q* products, is an important takeaway from Fig. 6c. Being flanked by the dominance of lateral spreading of elastic waves at low frequency (green points/line) and by surface scattering at higher frequencies (black dotted line), the *f* × *Q* product at the 100–200 GHz range exceeds that required for ground state laser cooling (*f* × *Q* = 6 × 10^12^) by more than order of magnitude. Data from the tri-layer cavity in Fig. 4 (blue stars in Fig. 6c) offers an example of how a relatively lossy material (h-BN) can be added judiciously to the acoustic device to expand functionality, while maintaining high overall cavity performance (see *f* × *Q* ≈ 3 × 10^13^ for the 200 GHz mode of the tri-layer). We view this approach as a path toward integrated optomechanical systems where the cavity couples high-frequency elastic waves to embedded quantum systems (e.g., optical emitters in h-BN), while the acoustic interaction with the quantum system can be further tuned using advanced methods of laser cooling^59^ or coherent phonon manipulation (e.g., refs. ^60–62^).

In summary, we have shown how heterogeneities within high-quality 2D material structures extend the functionality of acoustic cavities operating in the GHz to THz frequency range. We demonstrated differential readout for adjacent MoS_2_ cavities detuned by a monolayer step, as well as a frequency comb generator in a heterostructured h-BN/MoS_2_ cavity. By employing MoS_2_ as a thin transducer for h-BN, we successfully extracted the longitudinal phonon lifetimes for optically transparent h-BN. Strain engineering within tri-layer MoS_2_/h-BN/MoS_2_ laminar structures resulted in strongly coupled acoustic cavities with coupling strengths of Γ ≈ 47 GHz. Our first-principles theoretical analysis provides a benchmark for the highest attainable, material-limited performance for 2D acoustic cavities at different temperatures. We find that the currently available 2D material quality and fabrication techniques can produce acoustic devices that operate within a factor of three of their fundamental anharmonic limit at RT, where we measure *f* × *Q* products in excess of 1 × 10^14^ in vicinity of 100–200 GHz frequency range. We anticipate that by extending the palette of 2D materials and by invoking different types of heterogeneities within 2D acoustic cavities, numerous acoustic devices will become available for advanced phonon-based sensing and signal processing.

## Methods

### Sample fabrication

In order to mitigate extrinsic contributions to the acoustic losses, we fabricated 2D material structures with minimal exposure to wet chemistry agents. To fabricate samples, we first lithographically patterned and etched wells and trenches into a Si or SiO_2_/Si substrate (e.g., Fig. 1a, well diameter: 1–10 μm; well depth: 250–425 nm). Then a Ti (5 nm)/Au (40 nm) film was evaporated on the surface, which served two purposes: (i) facilitating the exfoliation of large-area samples (e.g., ref. ^63^) and (ii) to enhance the optical cavity formed between the bottom of the wells and the suspended films. Bulk MoS_2_ crystals were directly exfoliated onto these substrates, leaving behind suspended flakes ranging in thickness from monolayer to bulk (see Supplementary Fig. 1). Measurements were performed on flakes ranging in thickness from 2.5 nm (4 L) up to 60 nm. Heterostructures (h-BN/MoS_2_ and MoS_2_/h-BN/MoS_2_) were fabricated using a PPC/PDMS stamping technique^20^ where h-BN and MoS_2_ films were exfoliated onto different SiO_2_ substrates. The PPC/PDMS stamp was then used to sequentially pick up the predetermined layers. Afterwards the heterostructure stacks were thermally released on the well-etched substrate described above. To determine film thicknesses for MoS_2_ flakes under ten layers, we use a combination of optical microscopy and Raman spectroscopy (e.g., peak positions of the interlayer breathing and shear modes of MoS_2_^64^, see Supplementary Fig. 2). For MoS_2_ films >10 layers and for all h-BN layers we used atomic force microscopy (AFM) to measure film thickness.

### Pump-probe measurements

An ultrafast optical pump-probe setup with micrometer-scale spatial resolution was used to study longitudinal vibrations in both MoS_2_ and MoS_2_/h-BN suspended films. Two femtosecond Ti:Sapphire lasers with repetition rate ~960 MHz provided pump (750 nm wavelength, 3–10 mW power) and probe (830 nm, ≤1 mW) pulses with variable time delay governed by asynchronous optical sampling (ASOPS)^65,66^. The detuning between the pump and probe repetition rate was set at 2.5 kHz. Both beams were focused using 50× objective lens and independently positioned with respect to structures of interest. The same objective also provided optical imaging of the sample and was used to collect the reflected probe light.

In order to facilitate optical readout, the wells in the substrate were pre-etched to a depth that approximately equals one-half the wavelength of the probe beam (λ_probe_ ~830 nm), which forms an optical cavity between the suspended flake and bottom mirror. This configuration in combination with a high repetition rate allows us to use relatively low pulse energy in order to provide acceptable signal-to-noise ratio while mitigating overheating and ensuring linear response of the mechanical structure. Typical acquisition mode included 50,000 sweeps averaged over accumulation time of 20 s. All the measurements were done at RT in ambient conditions.

### Phonon lifetime calculations

For first-principles calculations, the strength of each scattering is determined by matrix elements built from third-order anharmonic perturbations, frequencies, and eigenvectors of the phonons^51,53,67^ derived from DFT. Phonon scattering due to point defects, such as isotopic variation, is negligible for the frequency range considered here, particularly around RT. This method has been found to give good agreement with measured data in a wide range of bulk materials for phonon dispersions^68–70^ and for thermal conductivities^67,71,72^, where the latter are governed by phonon scattering. Details are given in the Supplementary Notes 7, 8.

## Supplementary information

Supplementary Information

Description of Additional Supplementary Files

Supplementary Movie 1

Supplementary Movie 2

## Data Availability

The authors declare that the data supporting this study are available within the article and its Supplementary Information files. Further information is available from the corresponding authors upon reasonable requests.

## Code availability

Data for Figs. 5 and 6 were produced using publicly available software (Quantum Espresso). Relevant input and harmonic and anharmonic force constant files can be available upon request.

## References

[1] Wallucks A, Marinkovic I, Hensen B, Stockill R, Groblacher S (2020). A quantum memory at telecom wavelengths. Nat. Phys..

[2] Forsch M (2020). Microwave-to-optics conversion using a mechanical oscillator in its quantum ground state. Nat. Phys..

[3] Fang K, Matheny MH, Luan X, Painter O (2016). Optical transduction and routing of microwave phonons in cavity-optomechanical circuits. Nat. Photonics.

[4] Gertler S, Kharel P, Kittlaus EA, Otterstrom NT, Rakich PT (2020). Shaping nonlinear optical response using nonlocal forward Brillouin interactions. N. J. Phys..

[5] Shao LB (2019). Phononic band Structure Engineering for High-Q Gigahertz Surface Acoustic Wave Resonators on Lithium Niobate. Phys. Rev. Appl..

[6] Zhang ZZ (2020). Coherent phonon dynamics in spatially separated graphene mechanical resonators. Proc. Natl Acad. Sci. USA.

[7] Chan J (2011). Laser cooling of a nanomechanical oscillator into its quantum ground state. Nature.

[8] O’Connell AD (2010). Quantum ground state and single-phonon control of a mechanical resonator. Nature.

[9] Silveri MP, Tuorila JA, Thuneberg EV, Paraoanu GS (2017). Quantum systems under frequency modulation. Rep. Prog. Phys..

[10] Metcalfe M, Carr SM, Muller A, Solomon GS, Lawall J (2010). Resolved sideband emission of InAs/GaAs quantum dots strained by surface acoustic waves. Phys. Rev. Lett..

[11] Carter SG (2019). Tunable Coupling of a Double Quantum Dot Spin System to a Mechanical Resonator. Nano Lett..

[12] Ge S (2014). Coherent Longitudinal Acoustic Phonon Approaching THz Frequency in Multilayer Molybdenum Disulphide. Sci. Rep..

[13] Soubelet P (2019). The lifetime of interlayer breathing modes of few-layer 2H-MoSe2 membranes. Nanoscale.

[14] Jeong TY (2016). Coherent Lattice Vibrations in Mono- and Few-Layer WSe2. ACS Nano.

[15] Greener JDG (2018). Coherent acoustic phonons in van der Waals nanolayers and heterostructures. Phys. Rev. B.

[16] Anguiano S (2017). Micropillar Resonators for Optomechanics in the Extremely High 19-95-GHz Frequency Range. Phys. Rev. Lett..

[17] Lanzillotti-Kimura ND, Fainstein A, Balseiro CA, Jusserand B (2007). Phonon engineering with acoustic nanocavities: Theoretical considerations on phonon molecules, band structures, and acoustic Bloch oscillations. Phys. Rev. B.

[18] Fainstein A, Lanzillotti-Kimura ND, Jusserand B, Perrin B (2013). Strong Optical-Mechanical Coupling in a Vertical GaAs/AlAs Microcavity for Subterahertz Phonons and Near-Infrared Light. Phys. Rev. Lett..

[19] Ortíz O, Esmann M, Lanzillotti-Kimura ND (2019). Phonon engineering with superlattices: Generalized nanomechanical potentials. Phys. Rev. B.

[20] Wang L (2013). One-Dimensional Electrical Contact to a Two-Dimensional Material. Science.

[21] Rhodes D, Chae SH, Ribeiro-Palau R, Hone J (2019). Disorder in van der Waals heterostructures of 2D materials. Nat. Mater..

[22] Wakafuji Y (2020). 3D Manipulation of 2D Materials Using Microdome Polymer. Nano Lett..

[23] Auld B. A. Acoustic Fields and Waves in Solids. A Whiley-Interscience Publication (1973).

[24] Ruello P, Gusev VE (2015). Physical mechanisms of coherent acoustic phonons generation by ultrafast laser action. Ultrasonics.

[25] Peelaers H, Van de Walle CG (2012). Effects of strain on band structure and effective masses in MoS2. Phys. Rev. B.

[26] Babilotte P (2010). Femtosecond laser generation and detection of high-frequency acoustic phonons in GaAs semiconductors. Phys. Rev. B.

[27] Kafi F, Pilevar Shahri R, Benam MR, Akhtar A (2017). Tuning Optical Properties of MoS2 Bulk and Monolayer Under Compressive and Tensile Strain: A First Principles Study. J. Electron. Mater..

[28] COMSOL. https://www.comsol.com/structural-mechanics-module.

[29] Liu Y, Huang Y, Duan X (2019). Van der Waals integration before and beyond two-dimensional materials. Nature.

[30] Wang J (2019). Strong vibrational coupling in room temperature plasmonic resonators. Nat. Commun..

[31] Novotny L (2010). Strong coupling, energy splitting, and level crossings: A classical perspective. Am. J. Phys..

[32] Bruchhausen AE (2018). Acoustic confinement phenomena in oxide multifunctional nanophononic devices. Phys. Rev. Mater..

[33] Frisenda R (2018). Recent progress in the assembly of nanodevices and van der Waals heterostructures by deterministic placement of 2D materials. Chem. Soc. Rev..

[34] Cheng CT (2017). Frequency conversion with nonlinear graphene photodetectors. Nanoscale.

[35] Rose LRF, Vien BS, Chiu WK (2020). Analytical solutions for crack-like scatterers and sources in isotropic elastic plates. Wave Motion.

[36] Li C, Gusev V, Dekorsy T, Hettich M (2019). All optical control of comb-like coherent acoustic phonons in multiple quantum well structures through double-pump-pulse pump-probe experiments. Opt. Express.

[37] Li C, Gusev V, Dimakis E, Dekorsy T, Hettich M (2019). Broadband Photo-Excited Coherent Acoustic Frequency Combs and Mini-Brillouin-Zone Modes in a MQW-SESAM Structure. Appl. Sci..

[38] Zuo WL, Li P, Zhang JR, Fang YM (2016). Analytical modeling of thermoelastic damping in bilayered microplate resonators. Int J. Mech. Sci..

[39] Grossmann, M. et al. Optical generation of a broadband acoustic frequency comb in the 100 GHz-regime. IEEE (2013).

[40] Grossmann M (2013). Femtosecond spectroscopy of acoustic frequency combs in the 100-GHz frequency range in Al/Si membranes. Phys. Rev. B.

[41] Rooney AP (2017). Observing Imperfection in Atomic Interfaces for van der Waals Heterostructures. Nano Lett..

[42] Rokni H, Lu W (2020). Direct measurements of interfacial adhesion in 2D materials and van der Waals heterostructures in ambient air. Nat. Commun..

[43] Bosak A (2006). Elasticity of hexagonal boron nitride: Inelastic x-ray scattering measurements. Phys. Rev. B.

[44] Peelaers H, Van de Walle CG (2014). Elastic Constants and Pressure-Induced Effects in MoS2. The. J. Phys. Chem. C..

[45] Tran TT, Bray K, Ford MJ, Toth M, Aharonovich I (2016). Quantum emission from hexagonal boron nitride monolayers. Nat. Nanotechnol..

[46] Tran TT (2016). Quantum Emission from Defects in Single-Crystalline Hexagonal Boron Nitride. Phys. Rev. Appl..

[47] Grosso G (2017). Tunable and high-purity room temperature single-photon emission from atomic defects in hexagonal boron nitride. Nat. Commun..

[48] Exarhos AL, Hopper DA, Grote RR, Alkauskas A, Bassett LC (2017). Optical Signatures of Quantum Emitters in Suspended Hexagonal Boron Nitride. ACS Nano.

[49] Tucker, J. W. & Rampton, V. W. Microwave Ultrasonics in Solid State Physics. North-Holland Publishing Company, Americal Elsevier Publishing Company (1972).

[50] Mason, W. P. & Thurston, R. N Physical Acoustics Principles and Methods. Academic Press (1971).

[51] Paulatto L, Mauri F, Lazzeri M (2013). Anharmonic properties from a generalized third-order ab initio approach: Theory and applications to graphite and graphene. Phys. Rev. B.

[52] Chou TH (2019). Long mean free paths of room-temperature THz acoustic phonons in a high thermal conductivity material. Phys. Rev. B.

[53] Ziman J. M. Electrons and Phonons. Oxford at the Clarendon Press (1962).

[54] Liang JR (2019). Impact of Post-Lithography Polymer Residue on the Electrical Characteristics of MoS2 and WSe2 Field Effect Transistors. Adv. Mater. Interfaces.

[55] Woodruff TO, Ehrenreich H (1961). Absorption of Sound in Insulators. Phys. Rev..

[56] Photiadis DM (2020). Photoexcited elastic waves in free-standing GaAs films. Phys. Rev. B.

[57] Bresnehan MS (2014). Prospects of direct growth boron nitride films as substrates for graphene electronics. J. Mater. Res.

[58] Jang SK, Youn J, Song YJ, Lee S (2016). Synthesis and Characterization of Hexagonal Boron Nitride as a Gate Dielectric. Sci. Rep..

[59] MacQuarrie ER, Otten M, Gray SK, Fuchs GD (2017). Cooling a mechanical resonator with nitrogen-vacancy centres using a room temperature excited state spin-strain interaction. Nat. Commun..

[60] Mahboob I, Nishiguchi K, Okamoto H, Yamaguchi H (2012). Phonon-cavity electromechanics. Nat. Phys..

[61] Cho S (2018). Strong Two-Mode Parametric Interaction and Amplification in a Nanomechanical Resonator. Phys. Rev. Appl..

[62] Okamoto H (2013). Coherent phonon manipulation in coupled mechanical resonators. Nat. Phys..

[63] Huang Y (2020). Universal mechanical exfoliation of large-area 2D crystals. Nat. Commun..

[64] Zhao Y (2013). Interlayer Breathing and Shear Modes in Few-Trilayer MoS2 and WSe2. Nano Lett..

[65] Bartels A (2007). Ultrafast time-domain spectroscopy based on high-speed asynchronous optical sampling. Rev. Sci. Instrum..

[66] Laser Quantum, ASOPS Engine, https://www.laserquantum.com/products/detail.cfm?id=127.

[67] Lindsay L, Broido DA, Reinecke TL (2012). Thermal Conductivity and Large Isotope Effect in GaN from First Principles. Phys. Rev. Lett..

[68] Giannozzi P, De Gironcoli S, Pavone P, Baroni S (1991). Abinitio calculation of phonon dispersions in semiconductors. Phys. Rev. B.

[69] Baroni S, De Gironcoli S, Dal Corso A, Giannozzi P (2001). Phonons and related crystal properties from density-functional perturbation theory. Rev. Mod. Phys..

[70] Tornatzky H, Gillen R, Uchiyama H, Maultzsch J (2019). Phonon dispersion in MoS2. Phys. Rev. B.

[71] Broido DA, Malorny M, Birner G, Mingo N, Stewart DA (2007). Intrinsic lattice thermal conductivity of semiconductors from first principles. Appl. Phys. Lett..

[72] Lindsay L, Hua C, Ruan XL, Lee S (2018). Survey of ab initio phonon thermal transport. Mater. Today Phys..

