# Supplementary material for: Acoustic cavities in 2D heterostructures
Source: Nat Commun. 2021 Jun 1;12:3267. doi: 10.1038/s41467-021-23359-7 (PMC8169679; doi:10.1038/s41467-021-23359-7)
Supplement: Supplementary file 1 — Supplementary Information [file 41467_2021_23359_MOESM1_ESM.pdf]

Supplementary Information

Acoustic cavities in 2D heterostructures

Maxim K. Zalalutdinov[*1†], *Jeremy T. Robinson*[*1†], *Jose J. Fonseca*[2], *Samuel W. LaGasse*[2], *Tribhuwan Pandey*[3], *Lucas R. Lindsay*[4], *Thomas L. Reinecke*[1], *Douglas M. Photiadis*[1], *James C. Culbertson*[1], *Cory D. Cress*[1], *Brian H. Houston*[1]

[1]US Naval Research Laboratory, Washington DC 20375

[2]NRC Postdoctoral Fellow at Naval Research Laboratory, Washington DC 20375

[3]Department of Physics, University of Antwerp-B2020, Antwerp, Belgium

[4]Materials Science and Technology Division, Oak Ridge National Laboratory, Oak Ridge, TN 37831

*Corresponding Author; †Equal Contribution

Supplementary Figures

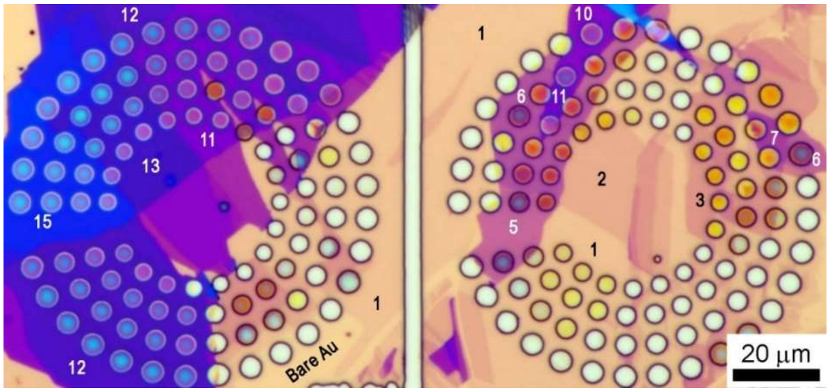

Supplementary Figure 1: Optical image. Two patterned regions from a sample where MoS_2 was exfoliated onto a Au/Ti coated substrate with pre-etched circular wells. A wide range of MoS_2 flake thicknesses are available for measurements. Several data points in Figure 1e of the main text were acquired from MoS_2 drums within this region.

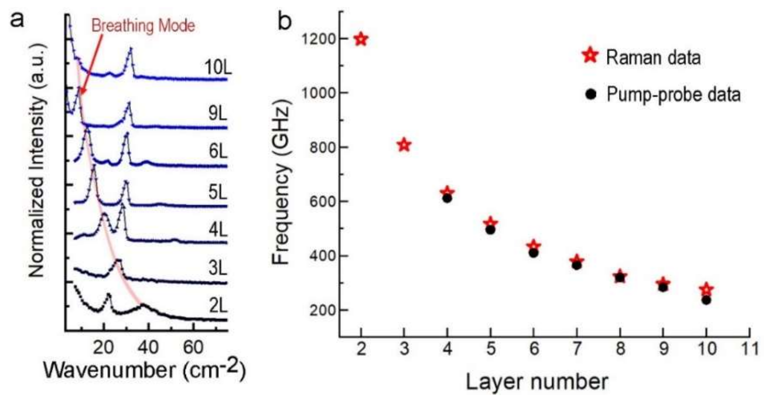

Supplementary Figure 2: Sample characterization. (a) Plot showing examples of the Raman breathing modes from MoS_2 flakes of several different thicknesses taken from the same sample as used for pump-probe experiments. Raman spectroscopy was used to obtain high confidence in layer identification for samples less than 10-layers thick. (b) Plot comparing the breathing mode frequencies measured using the ultrafast pump-probe setup discussed in the main text (black circles) to the frequencies of the breathing modes measured via Raman spectroscopy (red stars). Data points between the two different measurements are within 5% of each other.

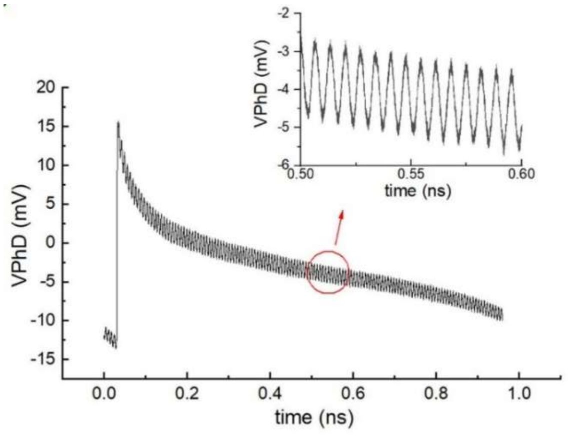

Supplementary Figure 3: Pump-Probe raw data. Output voltage of the balanced photodetector (V_{PhD}) is recorded, which measures the difference in light intensity between the reference arm of the probe beam and the probe light reflected from the sample: $V_{PhD} = G \times (I_{ref} - I_{sample})$. The acquisition time in the ASOPS system is converted to a delay between the pump and

probe pulses [1]. The moment when the pump and probe beams coincide at the sample is marked by a sharp change of reflectivity. The slow signal decay is attributed to carrier relaxation in MoS_2, while the fast oscillations of reflectivity (zoom-in shown in the inset) are caused by mechanical vibrations of the MoS_2 slab.

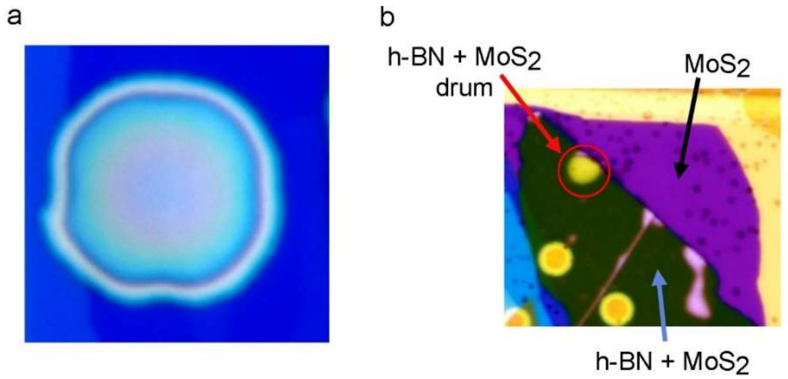

Supplementary Figure 4: Optical images. (a) The MoS_2 drum (10 μm diameter) with a monolayer step that was measured in Figure 2 of the main text and (b) the MoS_2/h-BN bi-layer structure measured in Figure 3 of the main text.

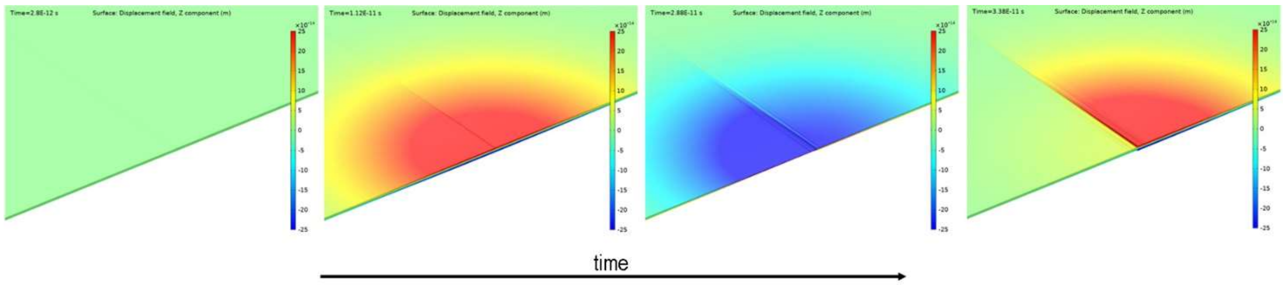

Supplementary Figure 5: Co-vibrating laterally-abutted, step-separated cavities. Snap-shots taken from the animation (see Supplementary Movie 1 and Supplementary Note 2), which shows time evolution of the out-of-plane displacement pattern for a MoS_2 plate excited by a sharply focused ($R_{1/e}$= 0.5 μm), ultrafast laser pulse. The MoS_2 plate shown here features a monolayer step that separates half-planes of 18L (left) and 19L (right) thicknesses. Comparison with more refined 2D FEM simulations (not shown) suggests that the ripples on the plate surface, which are visible in the movie, signify Lamb waves generated by the relative displacements of the abutted cavities at the monolayer step.

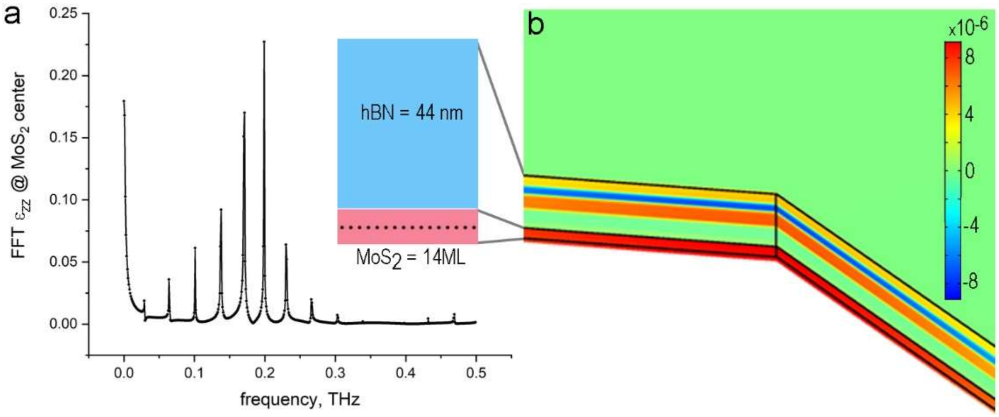

Supplementary Figure 6: Frequency Comb generator. The results of time-domain finite element modeling for the bilayer MoS_2/h-BN system. (a) FFT spectrum of the oscillating ε_{ZZ} strain component calculated for the point at the intersection of the MoS_2 layer's middle plane (dotted black line shown in MoS_2 in the inset) and the out-of-plane axis of symmetry. (b) A snapshot of the strain pattern: ε_{ZZ} component at 20ps after the initial pump pulse. The modeling approach is described in Supplementary Note 3.

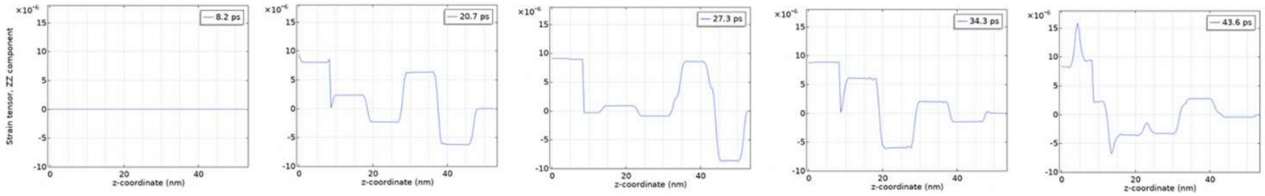

Supplementary Figure 7: Sound wave propagation in h-BN/MoS_2 bilayer. Snap-shots from the 1D animation (see Supplementary Movie 2) showing time evolution of the ε_{ZZ} component of the elastic strain in the h-BN(44nm)/ MoS_2 (14L or 8.7nm) bilayer along the axis of symmetry (central axis of the pump beam) for the first 50 picoseconds after the pump pulse. Examples of these strain profiles are shown in Figure 3d of the main text. The modeling approach is described in Supplementary Note 3.

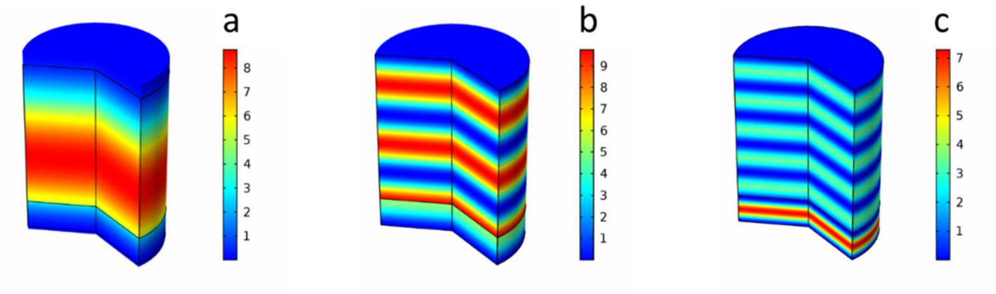

Supplementary Figure 8: Eigenmode analysis for h-BN/MoS_2 bilayer. FEM results for the elastic energy distribution in a 1D model of vibrating h-BN(44 nm, top)/ MoS_2(14L or 8.7 nm, bottom) bilayer structure. Modes at (a) 29 GHz, (b) 101 GHz, and (c) 199 GHz, matching the modes with the strain distributions depicted in Figure 3c of the main text are shown. Notably, the mode at 199 GHz (c) shows the energy distributions that is closest to the configuration of the energy deposited in the MoS_2 layer (bottom) by the pump laser beam. Accordingly, the amplitude of this mode in the vibrational spectrum (Figure 3c in the main text) is the highest. It is also one of the modes with the high MoS_2 partition index α_{MoS2} (see Supplementary Table 1 below).

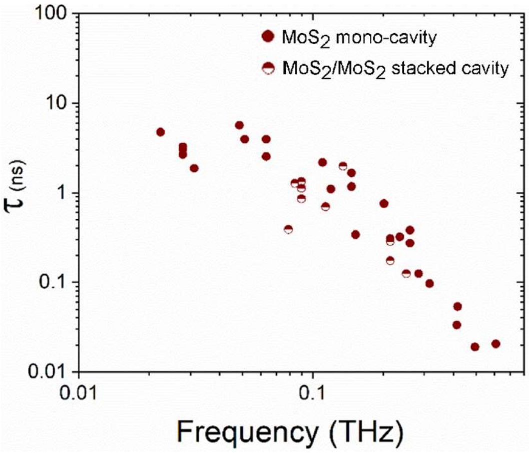

Supplementary Figure 9: Ring-down time measured for monolithic MoS_2 flakes and for stacked MoS_2/MoS_2 structures. The stacked cavities were fabricated by a sequential transfer of an additional MoS_2 flake on a top of a previously exfoliated MoS_2 flake, while the monolithic flakes were produced by a single exfoliation.

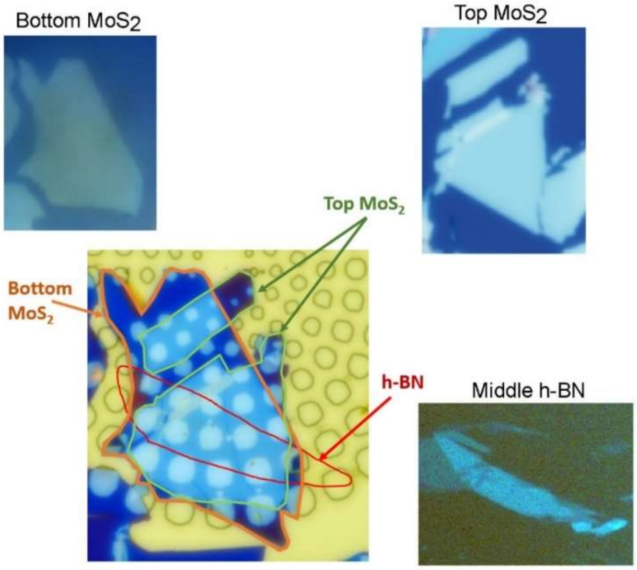

Supplementary Figure 10: Optical images. Individual flakes and completed stack for the MoS_2/h-BN/MoS_2 tri-layer structure are shown. Multiple data points were acquired from this region including drums comprised of: i) MoS_2/h-BN/MoS_2, ii) MoS_2/MoS_2 (bottom/top), and iii) MoS_2 alone (top or bottom). The measured frequency for the fundamental mode of the independent MoS_2 flakes provides high confidence in their layer thickness based on the thickness dependence plot in Figure 1 of the main text. The image contrast of the 'Middle h-BN' image was adjusted to better highlight the flake extent.

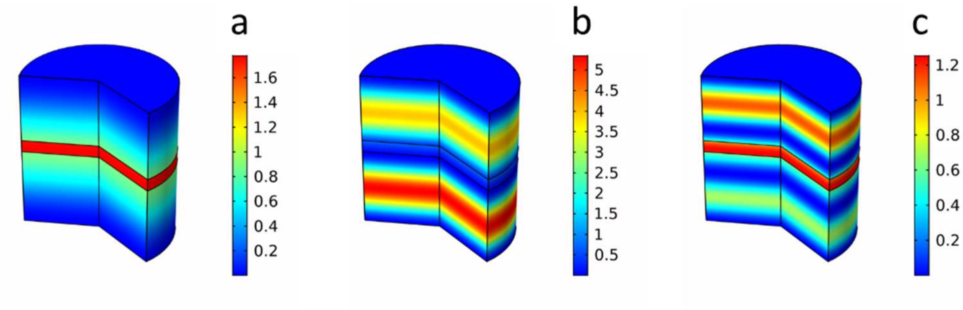

Supplementary Figure 11: Eigenmode analysis for MoS_2/h-BN/MoS_2 tri-layer. Elastic energy distribution in a 1D model of the vibrating MoS_2/h-BN/MoS_2 tri-layer structure. Modes at (a) 71 GHz, (b) 158 GHz, and (c) 216 GHz corresponding to the modes with the elastic strain depicted in Figure 3c of the main text are shown.

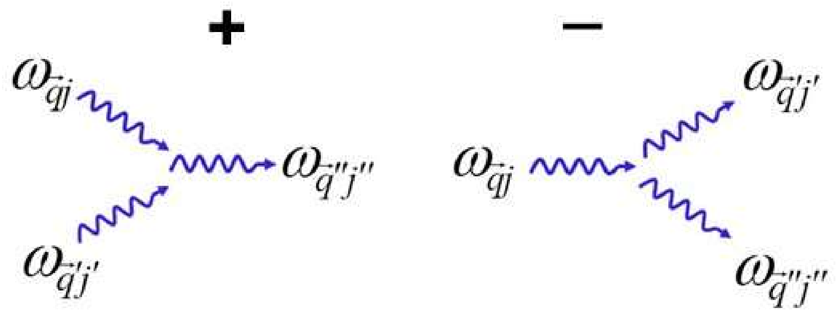

Supplementary Figure 12: **Three-phonon scattering.** Depiction of allowed three-phonon scattering processes with + corresponding to coalescence of two phonons into a third higher frequency phonon and – corresponding to phonon decay into two lower frequency phonons. The details of calculating phonon lifetimes are presented in Supplementary Notes 7, 8.

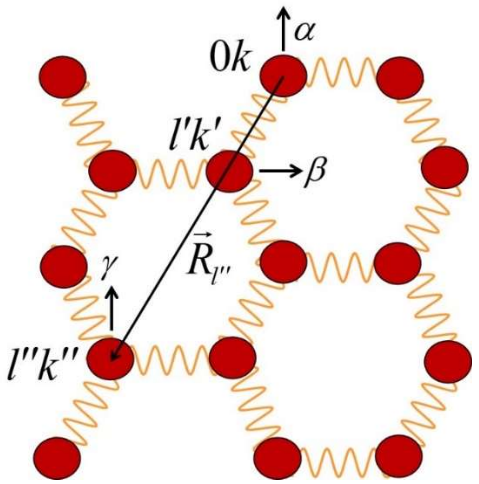

Supplementary Figure 13: **Cartoon depiction of a monolayer hexagonal lattice.** Atoms (red circles) are shown with nearest neighbor interatomic forces represented by orange springs. Small arrows represent atom perturbations required to calculate anharmonic interatomic force constants (IFC) $\Phi_{\alpha\beta\gamma}^{0k,l'k',l''k''}$.

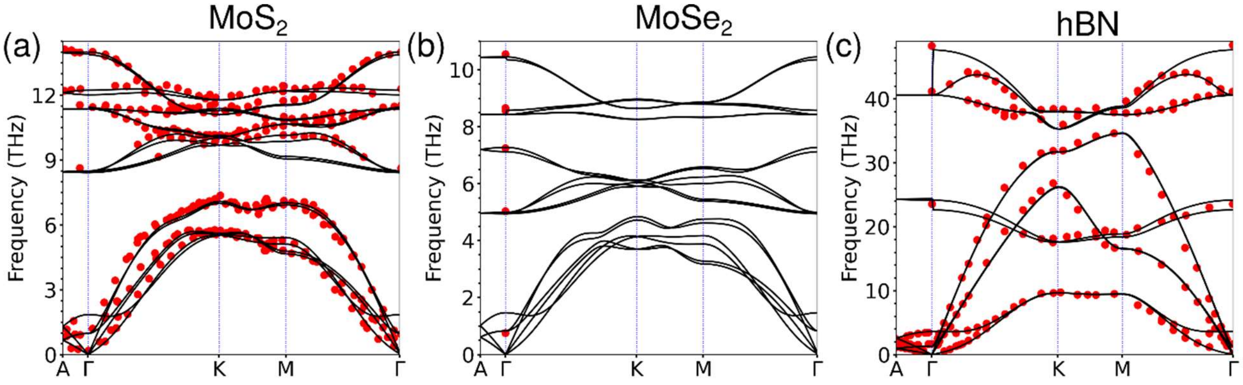

Supplementary Figure 14: **Calculated phonon dispersions.** Black lines show the results of calculations for the phonon dispersion in (a) MoS_2, (b) $MoSe_2$, and (c) h-BN. Red circles in (a) and (c) represent inelastic neutron scattering data from references [2] and [3], respectively. Measured $MoSe_2$ Raman data [4] is also shown in (b).

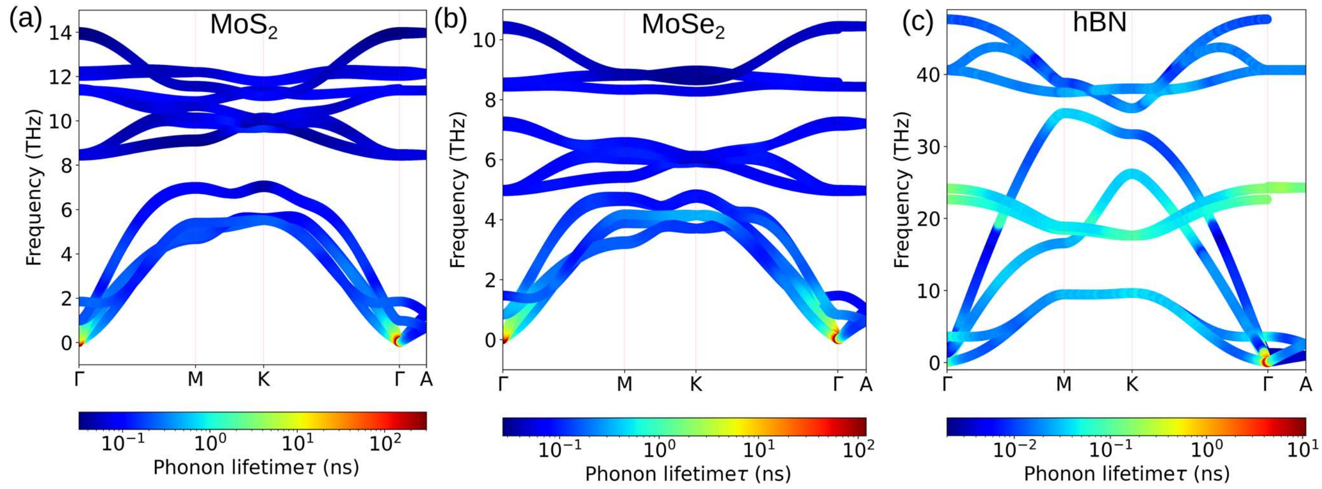

Supplementary Figure 15: **Phonon lifetimes projected on dispersion curves.** Anharmonicity-limited phonon lifetimes projected on the phonon dispersions for (a) MoS_2, (b) $MoSe_2$, and (c) h-BN.

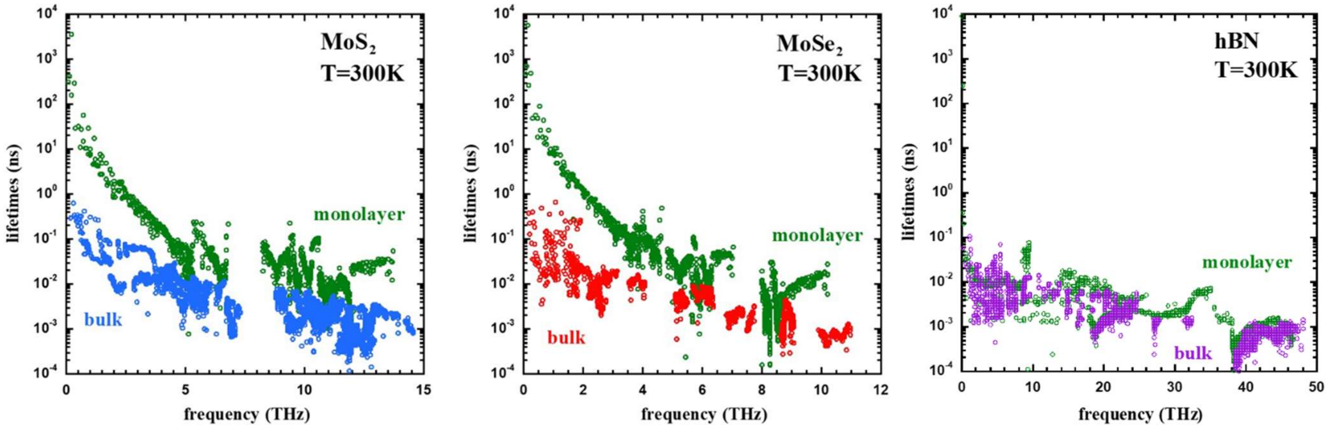

Supplementary Figure 16: Frequency dependence of the phonon lifetimes for different 2D materials. Phonon lifetimes through the full Brillouin zones and for all polarizations for (a) MoS_2, (b) $MoSe_2$, and (c) h-BN including three-phonon and phonon-isotope scatterings.

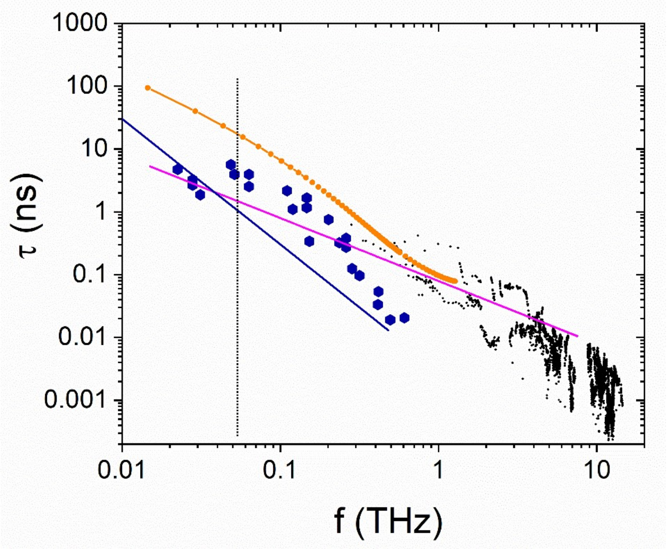

Supplementary Figure 17: **Model comparison for phonon lifetimes in MoS_2.** A comparison of *ab initio* calculations (orange and black points), experimental data (blue hexagons), and asymptotic theoretical models (Landau-Rumer, Akhiezer)[5]. The solid blue line shows the low frequency $\omega\tau_{th} \ll 1$ approximation (Akhiezer model) $\tau^{-1} = C_V T \gamma^2 \omega^2 \tau_{th} / 3\rho v^2$ [5]. The magenta line was produced by fitting 1/f dependence to the results of DFT calculations above 300 GHz and corresponds to Landau-Rumer frequency range $\omega\tau_{th} \gg 1$. Vertical dotted line marks the transition frequency $\omega\tau_{th} = 1$.

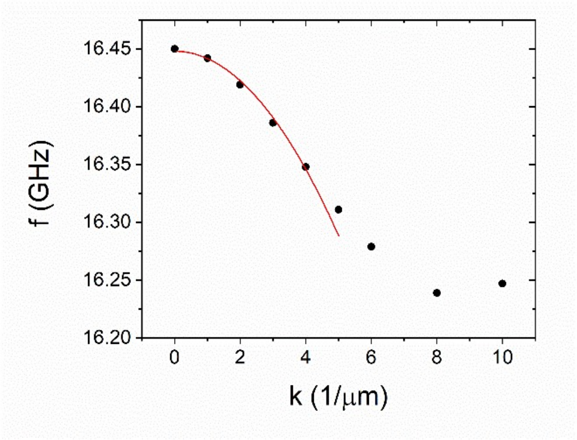

Supplementary Figure 18: **Lamb wave dispersion for a 160L-thick MoS_2 plate.** The approach outlined in Ref. [6] was used to calculate frequencies that correspond to given wave vectors using FEM eigenmode analysis. For every point shown in the graph, the analysis was done for a cut-out slice of the suspended plate and the modes corresponding to S1 Lamb waves were identified. The length of the slice (with periodic boundary conditions applied) defines the wave vector. The red line shows parabolic fit $\Delta f = Ak^2$.

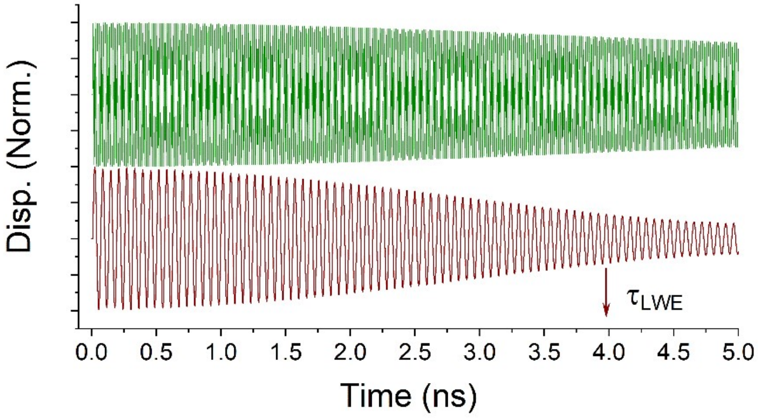

Supplementary Figure 19: **Lamb waves escape time in MoS_2 plates.** The results of time-domain FEM analysis are shown for the out-of-plane displacement (*i.e.*, dilation) under the center of the pump beam for a 40L MoS_2 plate (green) and 160L MoS_2 plate (maroon). The time interval, τ_{LWE}, counted from the pulse excitation to the point that shows threefold decrease in vibrations amplitude is interpreted as a Lamb waves escape time (τ_{LWE} shown by the arrow for the 160L film). The film thickness dependence of τ_{LWE} is shown in Figures 6a and 6c in the main text.

Supplementary Tables

Mode Frequency GHz	MoS_2 Energy fraction α_{MoS2}	h-BN Energy fraction α_{hBN}
29.5	0.03	0.97
64.26	0.07	0.93
101.16	0.1	0.90
137.6	0.16	0.84
170.54	0.29	0.71
199	0.28	0.72
230.44	0.14	0.86
266.42	0.09	0.91
303.41	0.11	0.89

Supplementary Table 1: Energy partitioning coefficients for different vibrational modes of h-BN (44nm, top)/ MoS_2 (14L or 8.7nm, bottom) bilayer structure, discussed in Figure 3 of the main text.

Mode Frequency GHz	MoS_2 Energy fraction α_{MoS2}	h-BN Energy fraction α_{hBN}
70.7	0.73	0.27
158	0.99	0.01
216	0.8	0.2

Supplementary Table 2: Energy partitioning coefficients for different vibrational modes of MoS_2(17ML)/h-BN(5ML)/MoS_2(15ML) tri-layer structure, discussed in Figure 4 of the main text.

	Lattice parameters		
	a (Å)	c (Å)	c/a
MoS_2	3.155 (3.160)	12.222 (12.296)	3.873 (3.891)
$MoSe_2$	3.282 (3.299)	12.839 (12.939)	3.912 (3.922)
h-BN	2.502 (2.505)	6.674 (6.653)	2.656 (2.667)

Supplementary Table 3: Calculated in-plane (a) and cross-plane (c) lattice parameters of MoS_2, $MoSe_2$, and h-BN. The values in the parenthesis are measured lattice parameters for MoS_2 and $MoSe_2$ [7] and for h-BN [8].

Supplementary Notes

Supplementary Note 1: Coupled oscillators model

In applying a 1DOF coupled oscillator model to the longitudinal vibrations of the MoS_2 step-cavities (Figure 2 main text) and tri-layer MoS_2/h-BN/MoS_2 (Figure 4 main text) systems, we follow the approach developed in Refs [9, 10]. Two oscillators are considered, such that in the absence of coupling would have eigenfrequencies $\omega_A^0 = \sqrt{k_A/m_A}$ and $\omega_B^0 = \sqrt{k_B/m_B}$. The equations of motion in the presence of coupling κ are given as:

$$m_A\ddot{x}_A + k_A x_A + \kappa(x_A - x_B) = 0 \quad (1)$$

$$m_B\ddot{x}_B + k_B x_B - \kappa(x_A - x_B) = 0 \quad (2)$$

The solution of the form $x_i(t) = x_i^0 \exp[-i\omega_\pm t]$ exists for mixed-mode frequencies

$$\omega_\pm^2 = \frac{1}{2}\left[\omega_A^2 + \omega_B^2 \pm \sqrt{(\omega_A^2 - \omega_B^2)^2 + 4\Gamma^2\omega_A\omega_B}\right] \quad (3)$$

where $\omega_A = \sqrt{(k_A + \kappa)/m_A}$, $\omega_B = \sqrt{(k_B + \kappa)/m_B}$, and Γ is anticrossing splitting:

$$\Gamma = \frac{\sqrt{\kappa/m_A}\sqrt{\kappa/m_B}}{\sqrt{\omega_A\omega_B}} \quad (4)$$

Abutted MoS_2 plates in the case of step-cavities (Figure 2 main text), as well as each of the outer MoS_2 layers in MoS_2/h-BN/MoS_2 tri-layer stack (Figure 4 main text) are considered as oscillators with the effective mass defined as

$$m_{A,B}^{eff} = A \int_{-\frac{h}{2}}^{\frac{h}{2}} \rho w(z)^2 dz \tag{5}$$

where $w(z) = \sin(\pi z/_h)$ is the eigenmode displacement, ρ is density, A is area, and h is the plate thickness [11].

By using the out-of-plane stiffness C33 of MoS_2 [12], the effective spring constants were assigned to each of the oscillators

$$k_{A,B} = \frac{A\pi^2}{2} \frac{C33}{h} \tag{6}$$

to match the resonant frequency of the fundamental thickness mode for corresponding plates in the uncoupled state (ω_A, ω_B).

<u>Abutted MoS_2 cavities separated by a monolayer step:</u>

For the abutted step-cavities, only an upper estimate is available for the coupling κ, extracted in the assumption that the splitting Γ does not exceed the half-width of the resonance, $\frac{f}{2Q}$. Such an estimate leads to $\kappa/_{k_A} \approx 2 \cdot 10^{-3}$.

Supplementary Note 2: Step-cavities, time-domain simulations

Time-dependent finite element modeling (FEM, COMSOL, Structural Mechanics Module [13]) was used to gain insights into the temporal behavior of strain patterns excited in the 2D acoustic hetero-cavities by the ultrafast pump pulses. The external strain option was used to mimic the film dilation, driven by photo-excited carriers and the deformation potential [14]. Both MoS_2 [12] and h-BN [15] films were modeled as fully anisotropic elastic solids.

In the Supplementary Movie 1, which examines the MoS_2 step-cavity system (Figure 2 of main text), the out-of-plane displacement is color coded and shows the "breathing" co-motion of the abutted step-cavities with 1L step height difference. The vibrations are activated using an

external strain pulse with a Gaussian shape both in time (pulse length τ= 500 fs) and in space (rad(1/e) = 0.5 μm in X-Y plane). For clarity, the higher overtones are suppressed by assigning cos$(\pi Z/h)$ thickness dependence to the external strain. The mesh is refined to include at least five elements across the total thickness (18L). A time step of 50 fs is chosen to ensure that the propagation range for longitudinal elastic waves in one step is much smaller than the element size. The geometry of the model is a circular plate (10μm in diameter) with the laser beam positioned at the center and the 1L step extending across the diameter. Due to the symmetry of the problem, the 3D model includes only half of the drum space, with the cut plane running orthogonal to the 1L step across the circle diameter. A symmetry condition was applied to the vertical cut plane. The spatial extent of the MoS_2 plate along the symmetry plane shown in the movie is 1.4 μm. Low reflecting boundary conditions are applied to outer edges of the plate (outside of the movie screen).

The pump-pulse in Supplementary Movie 1 peaks at 10 ps, while the plate is at rest at the start. Being spatially centered at the monolayer step, the pump provides equal excitation for both step-abutted cavities and initiates an *in-phase* dilation. Phase differences accumulate due to the difference in thickness (and therefore in eigenfrequency) and are visible at later stages in the movie.

Supplementary Note 3: Frequency comb, h-BN/MoS2 bilayer, time-domain analysis

The bilayer stack is modeled as suspended, while the perimeter treated as low-reflectivity boundary. External strain that imitates the effect of the pump-pulse is applied to the MoS_2 layer only. The spatial distribution for the "swelling" is Gaussian in the X-Y plane (rad(1/e) = 0.5 μm) and homogeneous across MoS_2 thickness (Z direction, normal to film surface). In an effort to reproduce temporal behavior of pump-generated strain, the external strain in the model is given a sharp rise ($\Delta t \approx 200$ fs) at the pulse arrival time ($t_0 = 10$ ps), followed by slow exponential decay ($\tau = 180$ ps [16]).

Supplementary Note 4: Energy dissipation in MoS_2/hBN laminates

The dissipation in our bilayer structure can be separated into individual contributions from h-BN and MoS_2 (see for example [17]):

$$\frac{1}{Q_{total}} = \frac{1}{2\pi}\frac{\Delta W_{hBN}+\Delta W_{MoS2}}{W_{hBN}+W_{MoS2}} = \frac{\Delta W_{hBN}}{2\pi W_{hBN}}\frac{W_{hBN}}{W_{hBN}+W_{MoS2}} + \frac{\Delta W_{MoS2}}{2\pi W_{MoS}}\frac{W_{MoS2}}{W_{hBN}+W_{MoS2}} \quad (7)$$

where W_{hBN} and W_{MoS2} are the energies stored in the corresponding layers and ΔW_{hBN} and ΔW_{MoS2} are layer-specific energy losses. FEM analysis readily provides coefficients for energy partitioning $\alpha_{hBN} = W_{hBN}/(W_{hBN} + W_{MoS2})$ and $\alpha_{MoS2} = W_{MoS2}/(W_{hBN} + W_{MoS2})$ (for example, $\alpha_{hBN} = 0.9$ and $\alpha_{MoS2} = 0.1$ for the 3rd overtone at 101 GHz, see Supplementary Figure 8 and Supplementary Table 1). Assuming that h-BN and MoS_2 layers are in equilibrium, the total ring-down time can be expressed as:

$$\frac{1}{\tau_{Total}} = \frac{\alpha_{hB}}{\tau_{hBN}} + \frac{\alpha_{MoS}}{\tau_{MoS2}} \quad (8)$$

Supplementary Equation 8 accounts for the fact that in an asymmetric bilayer structure (e.g. a monolayer of MoS_2 on top of a 100 nm thick plate of h-BN) the overall ring-down will be governed by the losses in the most-represented material (τ_{hBN}).

In our data analysis the bilayer ring-down time, τ_{Total}, was extracted first for each of the bilayer (h-BN/MoS_2) overtones by applying the corresponding band-pass FFT filters to the time-dependent reflectivity in Figure 3a in the main text. The relaxation time for boron nitride τ_{hBN} was then calculated from Supplementary Equation 8 using FEM-provided partition coefficients α and the experimentally measured, frequency-dependent MoS_2 ring-down time τ_{MoS2} (Figure 1, 6a in main text). We note that this procedure accounts for boundary-related contributions to energy dissipation in the MoS_2 layer (see Discussion in main text).

Supplementary Note 5: Material interfaces

An important practical issue arises from the fact that in vertical (laminar) heterocavities, the surface losses originate from both the outer free surfaces of the suspended slab, as well as from the inner materials' interfaces. In order to quantify the effect of these enclosed junctions (created by the sequential layer transfer) on the quality factor, *Q*, of the composite acoustic cavities, a series of samples were prepared by transferring MoS_2 layers on top of pre-existing suspended MoS_2 structures. It was critically important for this effort to find that the mechanical response of such stacked cavities comprised of a MoS_2/MoS_2 bilayer was indistinguishable from that of the monolithic MoS_2 cavities of an equal thickness prepared by a single exfoliation step. We note

this finding is in contrast with the behavior of stacked Au nanoparticles prepared by wet transfer, where the effect of boundary layer turned out to be prominent, as it provides significant mechanical isolation between the stacked nanoparticles [10].

For some MoS_2/MoS_2 stacked structures, the added inner interface did not have a distinguishable effect on the Q value of the cavity. For others, the Q value decreased by a factor of two. As the difference in performance between the MoS_2 stacked cavities and the mono-cavities appears close to the scatter within the mono-cavities themselves, we interpret this as strong evidence for the ability to build intricate 2D material acoustic structures where the interface plays a negligible role.

Supplementary Note 6: Coupled cavities in MoS_2/h-BN/MoS_2 tri-layer

Coupling, defined as $\kappa = Bk_A$ (where B is a constant), was used as a fitting parameter to match the calculated frequencies of mixed modes $\omega_\pm$ with the experimentally observed tri-layer overtones that correspond to the anti-symmetric (2nd overtone) and the symmetric (3rd overtone) strain distribution across the stack.

For the tri-layer stack the ratio $\kappa/k_A = B = 0.35$ with the corresponding anti-crossing splitting Γ = 47 GHz provided frequencies ω_+ = 215 GHz, ω_- = 162 GHz, in good agreement with experimental results for ω_2=158.8 GHz and ω_3 = 215 GHz (see Figure 4a in main text).

While interpreting the overtones of the MoS_2/h-BN/MoS_2 tri-layer as mixed modes ($\omega_\pm$, see Supplementary Equation 3) of two distinct MoS_2 cavities that are coupled by a h-BN layer, we note that even for the prototype described here the $\Delta\omega = \omega_+ - \omega_-$ frequency split is in the range accessible for state-of-the-art electro-optical modulators [18]. This opens possibilities for advanced techniques of coherent phonon manipulation, including parametric transfer [19] that would enable, for example, fast strain manipulation in the h-BN layer. We also emphasize that in contrast to EHF coupled systems implemented with solution-based gold nanoparticles, which are separated by a polymer layer [20], the MoS_2/h-BN/MoS_2 sample features a well-defined, single-crystal spacer of quantized thickness, and is residue free (within our detection limits). We anticipate these

features will be critical for low-temperature operation, where a high degree of control and low energy loss is necessary.

Supplementary Note 7: Phonon lifetime calculations

Phonon-phonon scattering: Lattice anharmonicity gives rise to phonon-phonon scattering, thus accounting for a variety of vibrational properties of materials including finite thermal conductivities, mechanical dissipation, and temperature-dependent linewidths/lifetimes. Taylor expansion of the crystal potential gives a lowest order anharmonic perturbation corresponding to three-phonon interactions [21, 22] (see Supplementary Figure 12).

Each scattering conserves energy and crystal momentum, and its transition probability is determined by Fermi's golden rule:

$$W^{\pm}_{\vec{q}j,\vec{q}'j',\vec{q}''j''} = \frac{2\pi}{\hbar}\left|\left\langle (n_{\vec{q}j}-1)(n_{\vec{q}'j'}\mp 1)(n_{\vec{q}''j''}+1)\middle|V_3^{\pm}\middle|n_{\vec{q}j}n_{\vec{q}'j'}n_{\vec{q}''j''}\right\rangle\right|^2 \delta(\hbar\omega_{\vec{q}j} \pm \hbar\omega_{\vec{q}'j'} - \hbar\omega_{\vec{q}''j''}) \tag{9}$$

where $\vec{q}$ and j label a phonon's wave vector and polarization, $\omega_{\vec{q}j}$ is the angular frequency, $n_{\vec{q}j}$ are the Bose equilibrium populations, and $V_3^{\pm}$ is the anharmonic perturbation constructed from anharmonic interatomic forces constants (IFCs) and creation and annihilation operators [21, 22]. After application of the perturbation and algebraic accounting of combinatorial processes the transition probabilities are given by [23]:

$$W^{+}_{\vec{q}j,\vec{q}'j',\vec{q}''j''} = \frac{\pi\hbar}{4N}\frac{|\Psi_{-\vec{q}j,-\vec{q}'j',\vec{q}''j''}|^2}{\omega_{\vec{q}j}\omega_{\vec{q}'j'}\omega_{\vec{q}''j''}}(n_{\vec{q}'j'} - n_{\vec{q}''j''})\delta(\omega_{\vec{q}j} + \omega_{\vec{q}'j'} - \omega_{\vec{q}''j''})\Delta(\vec{q} + \vec{q}' - (\vec{q}'' - \vec{G})) \tag{10}$$

$$W^{-}_{\vec{q}j,\vec{q}'j',\vec{q}''j''} = \frac{\pi\hbar}{4N}\frac{|\Psi_{-\vec{q}j,\vec{q}'j',\vec{q}''j''}|^2}{\omega_{\vec{q}j}\omega_{\vec{q}'j'}\omega_{\vec{q}''j''}}(n_{\vec{q}'j'} + n_{\vec{q}''j''} + 1)\delta(\omega_{\vec{q}j} - \omega_{\vec{q}'j'} - \omega_{\vec{q}''j''})\Delta(\vec{q} - \vec{q}' - (\vec{q}'' - \vec{G})) \tag{11}$$

where N is the number of unit cells in the system, $\vec{G}$ is a reciprocal lattice vector, and the scattering matrix elements are given by:

$$\Psi_{\vec{q}j,\vec{q}'j',\vec{q}''j''} = \sum_{k,l'k',l''k''}\sum_{\alpha\beta\gamma}\frac{\Phi^{0k,l'k',l''k''}_{\alpha\beta\gamma}}{\sqrt{m_k m_{k'} m_{k''}}}\epsilon^{\vec{q}j}_{k\alpha}\epsilon^{\vec{q}'j'}_{k'\beta}\epsilon^{\vec{q}''j''}_{k''\gamma}\, e^{i\vec{q}'\cdot\vec{R}_{l'}}e^{i\vec{q}''\cdot\vec{R}_{l''}} \tag{12}$$

where $\epsilon_{k\alpha}^{\vec{q}j}$ is the eigenvector of phonon $\vec{q}j$ for the αth Cartesian component of the kth atom in a unit cell, m_k is the isotopically averaged mass of the kth atom, $\vec{R}_l$ is the lattice vector locating the lth unit cell, and $\Phi_{\alpha\beta\gamma}^{0k,l'k',l''k''}$ are third-order anharmonic interatomic force constants linking atoms k , k', and k'' in the origin, l', and l'' unit cells, respectively, as depicted Supplementary Figure 13.

Summing over all possible three-phonon scatterings that conserve energy and momentum then gives the inverse phonon lifetime [21]:

$$1/\tau_{\vec{q}j} = \sum_{\vec{q}'j',\vec{q}''j''} W_{\vec{q}j,\vec{q}'j',\vec{q}''j''}^{+} + \frac{1}{2} W_{\vec{q}j,\vec{q}'j',\vec{q}''j''}^{-} \quad (13)$$

The 1/2 factor in the second term accounts for double counting of identical processes. The frequencies and eigenvectors that enter this quantum perturbation formalism are determined by diagonalization of the dynamical matrix:

$$D_{\alpha\beta}^{kk'}(\vec{q}) = \frac{1}{\sqrt{m_k m_{k'}}} \sum_{l'} \Phi_{\alpha\beta}^{0k,l'k'} e^{i\vec{q}\cdot\vec{R}_{l'}} \quad (14)$$

where $\Phi_{\alpha\beta}^{0k,l'k'}$ are harmonic IFCs. The harmonic and anharmonic IFCs are determined from density functional theory descriptions of the atomic interactions for each system as detailed in the next section. Calculations of the phonon dispersions of bulk MoS_2, $MoSe_2$, and h-BN are compared with measured data in Supplementary Figure 14.

Phonon-boundary scattering: Attenuation of phonons due to sample boundaries was modeled via the empirical formula [21]:

$$1/\tau_{\vec{q}j}^{boundary} = \frac{1-p_{q_z}}{1+p_{q_z}} \frac{|v_{\vec{q}jz}|}{h} \quad (15)$$

where $v_{\vec{q}jz} = \partial\omega_{\vec{q}j}/\partial q_z$ is the phonon velocity, h is sample thickness, z is the direction perpendicular to the sample surfaces, and p_{q_z} is the specularity parameter [21]:

$$p_{q_z} = e^{-(4\pi\eta/\lambda_z)^2} \quad (16)$$

Where $\lambda_z = 2\pi/q_z$ is the wavelength of the incident phonon and η is the root mean square (rms) roughness of the surfaces. To correlate our bulk calculations with the finite sample sizes from experiments we define $h = \lambda_z/2$.

Other scattering considerations: Phonon lifetimes can also be limited by scattering from higher order anharmonic processes (e.g., four-phonon scattering), isotopic mass variance, and from sample-specific extrinsic defects (e.g., vacancies, grain boundaries). Here we briefly comment on the potential impact of such mechanisms when comparing calculations (which do not consider these) with measurements (which do not quantify these).

Like the anharmonic scattering calculations discussed above, phonon-isotope scattering can be calculated fully using first principles within the context of quantum perturbation theory and density functional theory [24, 25]. Phonon-isotope scattering generally scales as ω^4, thus for the ultralow frequency range considered here this mechanism is unimportant, unlike the situation when considering lifetimes of high frequency optic phonons [26]. We verified that phonon-isotope scattering was negligible in the $\omega < 1$ THz frequency range for each material, and thus only include this scattering mechanism in Figures 5a and 5b of the main text, which considers the entire frequency spectrum of each material.

Similarly, other point defects are expected to have similar low frequency scattering behavior as phonon-isotope scattering, scaling as ω^4 [27, 28]. Thus, we expect that vacancies (which are prevalent on Sulfur sites in MoS_2 [29]) and other point defects will not play a significant role in the frequency range of our measurements.

Higher order anharmonic interactions have been shown to play an important role in limiting phonon lifetimes, even at room temperature, in strongly anharmonic materials [30, 31] and in materials where three-phonon scattering is especially weak [32]. Nonetheless, calculations have demonstrated that at room temperature for $\omega < 1$ THz four-phonon scattering is small compared to three-phonon scattering in a variety of materials [30, 31, 32, 33, 34].

Supplementary Note 8: Density functional theory and computational details

Low frequency scattering rates: The harmonic and anharmonic IFCs were computed within the framework of density functional perturbation theory (DFPT) [35] using the generalized gradient approximation as implemented in QUANTUM ESPRESSO [36, 37] and the D3Q package [38]. The optimized norm-conserving Vanderbilt (ONCV) pseudo-potentials [39, 40] with 14 valence electrons for Mo ($3s^2$ $3p^6$ $3d^4$ $4s^2$), 6 for S/Se ($3s^2$ $3p^4$), 3 for B ($2s^2$ $2p^1$), and 5 ($2s^2$ $2p^3$) for N were used. The atomic positions and cell parameters were fully optimized until the forces between atoms were smaller than 10^{-5} Ry/Bohr. During structure relaxation the interlayer van der Waals (vdW) interactions were incorporated through the semi-empirical DFT-D3 correction with Becke-Johnson damping [41], which has been shown to reproduce measured lattice parameters and interlayer distances [2, 42]. A comparison between calculated and measured lattice parameters is given in Supplementary Table 3. Electronic k-point grids of 12 × 12 × 4 for MoS_2 and $MoSe_2$ and 15 × 15 × 6 for h-BN along with a wave-function cutoff of 120 Ry were used for Brillouin zone integrations. An extremely restrictive energy tolerance of 10^{-16} Ry was used for the starting ground state density and wave functions for the DFPT calculations.

For MoS_2 and $MoSe_2$ the harmonic and anharmonic IFCs were computed on 5×5×2 and 4×4×2 q-point grids, respectively. For h-BN the harmonic and anharmonic IFCs were computed on 6×6×3 and 5×5×3 grids, respectively. These IFCs were used to determine phonon lifetimes as limited by three-phonon interactions via quantum perturbation theory, i.e., Fermi's golden rule [21]. Gaussian smearing of 10 cm^{-1} was used to approximate the energy conserving delta functions for three-phonon interactions. Here, Brillouin zone integrations were performed with a uniform q-point grid of 108×108×36 for MoS_2/$MoSe_2$ and 100×100×40 for hBN. These calculations were used to characterize the low frequency phonon behaviors presented in Figures 5c, 5d, and 6 of the main text and Supplementary Figures 14-16.

Full Brillouin zone scattering rates: Monolayer calculations of the harmonic and anharmonic IFCs were determined by supercell calculations using the local density approximation implemented in QUANTUM ESPRESSO [36, 37] with norm conserving pseudopotentials: Martin-Troullier Perdew-Wang for Mo, S, and Se; Bachelet-Hamman-Schluter Perdew-Zunger for B; von Barth-Carr Perdew-Zunger for N. The atomic positions and cell parameters were optimized until the forces between atoms were smaller than 10^{-10} Ry/Bohr. Electronic k-point grid 13 × 13

× 1 and wave-function cutoff of 80 Ry were used for monolayer MoS_2 and $MoSe_2$, while a 16 × 16 × 1 grid and 120 Ry cutoff were used for monolayer h-BN. Energy tolerances of 10^{-12} Ry and 10^{-13} Ry were used for the starting ground state density and wave functions for $MoS_2/MoSe_2$ and h-BN, respectively. Vacuum spaces of 120 Å and 55 Å between layers in $MoS_2/MoSe_2$ and h-BN, respectively, were used to avoid spurious layer interactions. These DFT parameters gave lattice constants a=2.489 Å for monolayer h-BN, a=3.091 Å for MoS_2, and a=3.322 Å for $MoSe_2$. The calculated S-S and Se-Se layer distances were 3.096 Å and 3.352 Å, respectively, while monolayer h-BN is flat.

For monolayer MoS_2 and $MoSe_2$ the harmonic and anharmonic IFCs were computed with forces calculated from perturbed 243 atom supercells with Γ-point-only sampling. Nearest neighbor interactions were included to 13th and 5th shells, respectively. For monolayer h-BN 162 atom supercells were used including 13th and 8th nearest neighbor shell interactions. For comparison, similar LDA bulk calculations were performed for MoS_2 and h-BN as described in Ref. [43] and Ref. [44], respectively. Bulk $MoSe_2$ simulations were performed similar to those of MoS_2. These calculations were used to characterize all phonon polarizations throughout the Brillouin zones of each system as presented in Figs. 5a and 5b of the main text and Supplementary Figure 16 here.

Supplementary Note 9: Lateral spreading of the photogenerated elastic waves

One strategy to increase the density of elastic energy within the 2D acoustic cavity is to shrink the cavity footprint. This is accomplished by employing sharply focused laser beams for exciting the vibrations. As the spot size of the diffraction-limited pump beam becomes comparable to the film thickness, the in-plane evolution of elastic waves gains significance. Theoretical analysis [45] shows that spatial variation of the photo-induced elastic strain (defined by the intensity profile of the pump beam) gives rise to symmetric S1 Lamb waves with in-plane wavevectors $k_L \leq 2/R_{beam}$ that propagate outward, taking out the energy stored in the cavity. The energy leak rate can therefore be governed by the group velocity of the elastic waves (as opposed to internal friction) and is defined by the Lamb waves' dispersion law. In the vicinity of the thickness mode resonance, i.e. in the limit $k \rightarrow 0$ the S1 wave dispersion exhibits parabolic nature $\omega \sim k^2$ (see Supplementary Note 10), leading to group velocity proportional to film thickness:

$$v_{gr} = \frac{\partial \omega}{\partial k} \sim \frac{h}{R_{beam}} \tag{17}$$

A parabolic fit to the FEM-calculated dispersion law [6] in MoS_2 plates (Supplementary Figure 18) provides a value of $v_{gr}(k_L = 2/R_{beam}) \approx 320$ m/s for a 160L-thick film. The resulting escape time estimated as $\tau = 2R_{beam}/v_{gr} \approx 3$ ns is consistent with the ring-down time measured for thicker films in Figure 6a of the main text and shows that elastic wave spreading can be a factor affecting cavity performance.

In order to get a more quantitative assessment for the escape time, we use time domain simulations that explicitly model the MoS_2 plate response to a pulse-like elastic excitation (Supplementary Figure 19). The validity of this approach for estimating the Lamb waves escape time is limited to structures featuring optical penetration depth that greatly exceeds the film thickness, as the symmetric S1 mode is expected to dominate the vibrational spectrum under that condition.

We note that even though the estimates accounting for the lateral spreading of elastic energy agree well with our experimental data for relaxation times measured in MoS_2 cavities in sub-100 GHz frequency range, extra effort is needed to make this comparison fully quantitative. A different experimental approach (*e.g.*, similar to that described in [46]) might be required in order to circumvent the timing limitations of our pump-probe setup. The accuracy of measuring ring-down times in excess of 1 ns is convoluted by the high repetition rate $f_{rep} \approx 1$ GHz, which as τ becomes longer can cause a partial overlap of multiple slowly-decaying traces generated by preceding pump pulses.

Supplementary Note 10: Lamb wave dispersion

Consider the dispersion relation for the symmetric Lamb modes [47],

$$\frac{\tan(q_T h/2)}{\tan(q_L h/2)} = -\frac{4k^2 q_L q_T}{\left(q_T^2 - k^2\right)^2} \tag{18}$$

where h = layer thickness, and k magnitude of the wavenumber parallel to the layer surface. The notation is:

$k_T = \frac{\omega}{c_T}$ magnitude of the wavenumber of shear waves

$k_L = \frac{\omega}{c_L}$ magnitude of the wavenumber of longitudinal waves

$q_T = \sqrt{k_T^2 - k^2}$ wavenumber of shear wave perpendicular to layer surface

$q_L = \sqrt{k_L^2 - k^2}$ wavenumber of longitudinal wave perpendicular to layer surface

ω - angular frequency

To obtain the solutions $\omega(k)$ near $k = 0$, firstly, expand the dispersion relation in powers of k^2 to obtain,

$$\left(\tan k_T\, h/2 - \frac{k^2 h}{4k_T}\, sec^2 k_T\, h/2\right)\left(\cot k_L\, h/2 + \frac{k^2 h}{4k_L}\, csc^2 k_L\, h/2\right) \approx -\frac{4k^2 k_L}{k_T^3} \qquad (19)$$

where we omit terms of order k^4 and higher. With k_T and k_L proportional to ω, a solution (albeit a complicated one) can immediately be written for k in terms of a transcendental function of ω. The solutions can be grouped into quasi-longitudinal solutions ω_{nL} and quasi-transverse solutions ω_{nT}. We seek an analytical approximation to the inverse solutions $\omega_{nL}(k)$. Such a solution was given explicitly for the modes $\omega_{1L(T)}(k)$ in the case of near degeneracy of the modes $\omega_{1L}(k)$ and $\omega_{1T}(k)$.

To obtain an analytical solution in general, we follow the same procedure as regards ω: let $\omega = \omega_{nL} + \delta\omega$ where

$$\omega_{nL} = \frac{(2n-1)\pi c_L}{h} \qquad (20)$$

is the eigenfrequency for the nth longitudinal solution for $k = 0$. To consistently retain terms of order k^2 we must retain terms of order $\delta\omega$, $\delta\omega^2$ (in addition to those of order k^2) because the $\delta\omega$ may be proportional to either k or k^2. Define,

$$k_{nL} = \left.\frac{k_L h}{2}\right|_{\omega=\omega_{nL}} = (n - 1/2)\pi\,, \qquad (21)$$

$$k_{nT} = \left.\frac{k_T h}{2}\right|_{\omega=\omega_{nL}} = \frac{c_L}{c_T}(n - 1/2)\pi = \alpha(n - 1/2)\pi \qquad (22)$$

where $\alpha = c_L/c_T$, the ratio of longitudinal to shear wave speeds. Then we approximate the trigonometric functions appearing in the above dispersion relation as

$\tan(k_T h/2) \approx \tan\kappa_{nT} + \alpha\delta\omega_d \sec^2 \kappa_{nT},$
$\cot(k_L h/2) \approx -\delta\omega_d,$
$\sec(k_T h/2) \approx \sec(\kappa_{nT}),$
$\csc(k_L h/2) \approx (-1)^{n+1},$

where $\delta\omega_d = \frac{h\,\delta\omega}{2c_L}$ is a dimensionless version of the small change in frequency $\delta\omega$. Here we have noted that $\sin(\kappa_{nL}) = (-1)^{n+1}$ and $\cos(\kappa_{nL}) = 0$. We have also retained only leading order in the sec and csc expansions because of the presence of the factors of k^2 in Supplementary Equation 19 above. Then, substituting the trigonometric expansions into the approximate dispersion relations, (Supplementary Equation 19), we obtain to order k^2,

$$\delta\omega_d tan(k_{nT}) + \alpha\delta\omega_d^2 sec^2(k_{nT}) - \frac{k^2h^2}{4m\pi}\left(tan(k_{nT}) + \frac{16}{m\pi^{\ 3}}\right) = 0 \qquad (21)$$

with $m = 2n - 1$, an odd integer. This result is valid for all symmetric, longitudinal Lamb modes.

For sufficiently small $\delta\omega$ (and correspondingly k) one may observe that the dispersion relation is always quadratic in k. The curvature may be positive, negative, or nearly vanish depending on the quantity κ_{nT} and α and this in turn is closely related to the degeneracy of S_{1L} and S_{1T} modes.

We note that the considerations above are given for Lamb waves in isotropic materials. However, our numerical calculations (see example of dispersion curve in Supplementary Figure 18) indicate that the main features – parabolic dispersion at $k \to 0$ and linear scaling with the film thickness h are preserved for transversely isotropic materials, such as MoS_2.

Supplementary References

1. Bartels A*, et al.* Ultrafast time-domain spectroscopy based on high-speed asynchronous optical sampling. *Review of Scientific Instruments* **78**, 035107 (2007).

2. Tornatzky H, Gillen R, Uchiyama H, Maultzsch J. Phonon dispersion in MoS2. *Physical Review B* **99**, 144309 (2019).

3. Serrano J*, et al.* Vibrational properties of hexagonal boron nitride: Inelastic X-ray scattering and ab initio calculations. *Physical Review Letters* **98**, 095503 (2007).

4. Sekine T, Izumi M, Nakashizu T, Uchinokura K, Matsuura E. RAMAN-SCATTERING AND INFRARED REFLECTANCE IN 2H-MOSE2. *J Phys Soc Jpn* **49**, 1069-1077 (1980).

5. Woodruff TO, Ehrenreich H. ABSORPTION OF SOUND IN INSULATORS. *Physical Review* **123**, 1553-1559 (1961).

6. Hakoda C, Rose J, Shokouhi P, Lissenden C. Using Floquet periodicity to easily calculate dispersion curves and wave structures of homogeneous waveguides. In: *44th Annual Review of Progress in Quantitative Nondestructive Evaluation, Volume 37*). AIP Conference Proceedings 1949, 020016 (2018).

7. Hilli AAA, Evans BL. PREPARATION AND PROPERTIES OF TRANSITION-METAL DICHALCOGENIDE SINGLE-CRYSTALS. *J Cryst Growth* **15**, 93-101 (1972).

8. Paszkowicz W, Pelka JB, Knapp M, Szyszko T, Podsiadlo S. Lattice parameters and anisotropic thermal expansion of hexagonal boron nitride in the 10-297.5 K temperature range. *Appl Phys A-Mater Sci Process* **75**, 431-435 (2002).

9. Novotny L. Strong coupling, energy splitting, and level crossings: A classical perspective. *American Journal of Physics* **78**, 1199-1202 (2010).

10. Wang J, Yu K, Yang Y, Hartland GV, Sader JE, Wang GP. Strong vibrational coupling in room temperature plasmonic resonators. *Nature Communications* **10**, 1527 (2019).

11. Hauer BD, Doolin C, Beach KSD, Davis JP. A general procedure for thermomechanical calibration of nano/micro-mechanical resonators. *Ann Phys* **339**, 181-207 (2013).

12. Peelaers H, Van de Walle CG. Elastic Constants and Pressure-Induced Effects in MoS2. *The Journal of Physical Chemistry C* **118**, 12073-12076 (2014).

13. COMSOL. https://www.comsol.com/structural-mechanics-module.).

14. Ruello P, Gusev VE. Physical mechanisms of coherent acoustic phonons generation by ultrafast laser action. *Ultrasonics* **56**, 21-35 (2015).

15. Bosak A, Serrano J, Krisch M, Watanabe K, Taniguchi T, Kanda H. Elasticity of hexagonal boron nitride: Inelastic x-ray scattering measurements. *Physical Review B* **73**, 041402(R) (2006).

16. Kumar N, He J, He D, Wang Y, Zhao H. Charge carrier dynamics in bulk MoS2 crystal studied by transient absorption microscopy. *Journal of Applied Physics* **113**, (2013).

17. Zuo WL, Li P, Zhang JR, Fang YM. Analytical modeling of thermoelastic damping in bilayered microplate resonators. *Int J Mech Sci* **106**, 128-137 (2016).

18. Wang XX, Weigel PO, Zhao J, Ruesing M, Mookherjea S. Achieving beyond-100-GHz large-signal modulation bandwidth in hybrid silicon photonics Mach Zehnder modulators using thin film lithium niobate. *APL Phontonics* **4**, 096101 (2019).

19. Okamoto H*, et al.* Coherent phonon manipulation in coupled mechanical resonators. *Nature Physics* **9**, 480-484 (2013).

20. Wang JZ, Yang Y, Wang N, Yu K, Hartland GV, Wang GP. Long Lifetime and Coupling of Acoustic Vibrations of Gold Nanoplates on Unsupported Thin Films. *J Phys Chem A* **123**, 10339-10346 (2019).

21. Ziman JM. *Electrons and Phonons*. Oxford at the Clarendon Press (1962).

22. Srivastava GP. *The Physics of Phonons*. Adam Hilger (1990).

23. Polanco LLaCA. Thermal transport by first-principles anharmonic lattice dynamics. In: *Handbook of Materials Modeling* (ed Yip WAaS). Springer (2020).

24. Tamura S. ISOTOPE SCATTERING OF LARGE-WAVE-VECTOR PHONONS IN GAAS AND INSB - DEFORMATION-DIPOLE AND OVERLAP-SHELL MODELS. *Physical Review B* **30**, 849-854 (1984).

25. Lindsay L, Broido DA, Reinecke TL. Phonon-isotope scattering and thermal conductivity in materials with a large isotope effect: A first-principles study. *Physical Review B* **88**, 144306 (2013).

26. Giles AJ*, et al.* Ultralow-loss polaritons in isotopically pure boron nitride. *Nature Materials* **17**, 134-140 (2018).

27. Klemens PG. THE SCATTERING OF LOW-FREQUENCY LATTICE WAVES BY STATIC IMPERFECTIONS. *Proceedings of the Physical Society of London Section A* **68**, 1113-1128 (1955).

28. Polanco CA, Lindsay L. Thermal conductivity of InN with point defects from first principles. *Physical Review B* **98**, 014306 (2018).

29. Hong JH*, et al.* Exploring atomic defects in molybdenum disulphide monolayers. *Nature Communications* **6**, 6293 (2015).

30. Ravichandran NK, Broido D. Unified first-principles theory of thermal properties of insulators. *Physical Review B* **98**, 085205 (2018).

31. Xia Y. Revisiting lattice thermal transport in PbTe: The crucial role of quartic anharmonicity. *Applied Physics Letters* **113**, 073901 (2018).

32. Feng TL, Lindsay L, Ruan XL. Four-phonon scattering significantly reduces intrinsic thermal conductivity of solids. *Physical Review B* **96**, 161201(R) (2017).

33. Gu XK, Fan ZY, Bao H, Zhao CY. Revisiting phonon-phonon scattering in single-layer graphene. *Physical Review B* **100**, 064306 (2019).

34. Ravichandran NK, Broido D. Phonon-Phonon Interactions in Strongly Bonded Solids: Selection Rules and Higher-Order Processes. *Physical Review X* **10**, 021063 (2020).

35. Baroni S, de Gironcoli S, Dal Corso A, Giannozzi P. Phonons and related crystal properties from density-functional perturbation theory. *Reviews of Modern Physics* **73**, 515-562 (2001).

36. Giannozzi P*, et al.* Advanced capabilities for materials modelling with QUANTUM ESPRESSO. *J Phys-Condes Matter* **29**, 465901 (2017).

37. Giannozzi P*, et al.* QUANTUM ESPRESSO: a modular and open-source software project for quantum simulations of materials. *J Phys-Condes Matter* **21**, 395502 (2009).

38. Paulatto L, Mauri F, Lazzeri M. Anharmonic properties from a generalized third-order ab initio approach: Theory and applications to graphite and graphene. *Physical Review B* **87**, 214303 (2013).

39. Hamann DR. Optimized norm-conserving Vanderbilt pseudopotentials. *Physical Review B* **88**, 085117 (2013).

40. Schlipf M, Gygi F. Optimization algorithm for the generation of ONCV pseudopotentials. *Comput Phys Commun* **196**, 36-44 (2015).

41. Grimme S, Ehrlich S, Goerigk L. Effect of the Damping Function in Dispersion Corrected Density Functional Theory. *J Comput Chem* **32**, 1456-1465 (2011).

42. Tyborski C*, et al.* Electronic and Vibrational Properties of Diamondoid Oligomers. *J Phys Chem C* **121**, 27082-27088 (2017).

43. Polanco CA, Pandey T, Berlijn T, Lindsay L. Defect-limited thermal conductivity in MoS2. *Physical Review Materials* **4**, 014004 (2020).

44. Jiang P, Qian X, Yang R, Lindsay L. Anisotropic thermal transport in bulk hexagonal boron nitride. *Physical Review Materials* **2**, 064005 (2018).

45. Photiadis DM, Zalalutdinov MK, Bracker AS, Carter SG, Gammon D, Houston BH. Photoexcited elastic waves in free-standing GaAs films. *Physical Review B* **101**, 245304 (2020).

46. Bruchhausen A*, et al.* Subharmonic resonant optical excitation of confined acoustic modes in a free-standing semiconductor membrane at GHz frequencies with a high-repetition-rate femtosecond laser. *Phys Rev Lett* **106**, 077401 (2011).

47. Auld BA. *Acoustic Fields and Waves in Solids*. A Whiley-Interscience Publication (1973).
