# Supplementary material for: Acoustic cavities in 2D heterostructures
Source: Nat Commun. 2021 Jun 1;12:3267. doi: 10.1038/s41467-021-23359-7 (PMC8169679; doi:10.1038/s41467-021-23359-7)
Supplement: Supplementary file 2 — Description of Additional Supplementary Files [file 41467_2021_23359_MOESM2_ESM.docx]

**Description of Additional Supplementary Files**

**Supplementary Movie 1:** Step-cavity ring-down. Temporal response (normal displacement δz is color-coded) of MoS2 plate with a monolayer step (thickness 18/19ML) to a pulsed excitation generated by a step-centred beam.

**Supplementary Movie 2:** Elastic strain reverberating in a bilayer. Time evolution of the normal strain (εzz component) across the thickness of MoS2(8.7nm)/h-BN(44nm) heterostructures, under the centre of the pump beam. The initial strain originates from a selective pulsed excitation limited to MoS2 layer.
