# Supplementary figures and images for: Acoustic cavities in 2D heterostructures
Source: Nat Commun. 2021 Jun 1;12:3267. doi: 10.1038/s41467-021-23359-7 (PMC8169679; doi:10.1038/s41467-021-23359-7)

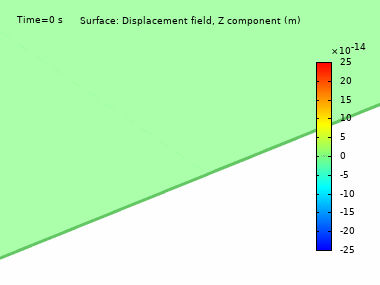

Supplement: Supplementary file 3 — Supplementary Movie 1 [file 41467_2021_23359_MOESM3_ESM.gif]

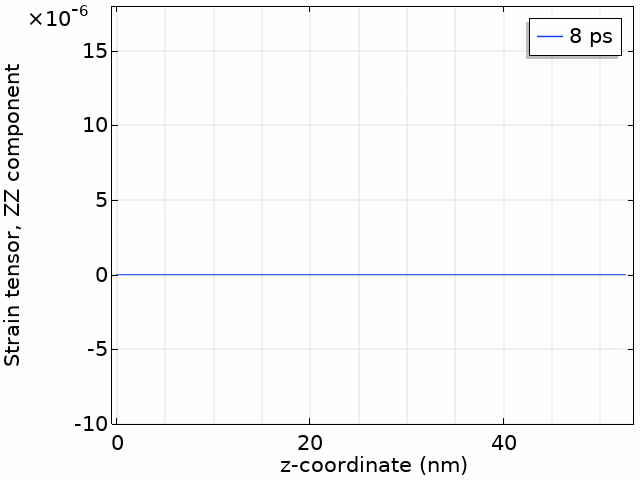

Supplement: Supplementary file 4 — Supplementary Movie 2 [file 41467_2021_23359_MOESM4_ESM.gif]
